# Performance of referral strategies for patients with suspected axial spondyloarthritis: a systematic review with meta-analysis

**DOI:** 10.1016/j.eclinm.2026.104167

**Published:** 2026-08-26

**Authors:** Daniel Feller, Roel Wingbermühle, Bart Koes, Sofia Ramiro, Alessandro Chiarotto

**Affiliations:** a Department of General Practice, Erasmus MC, University Medical Center, Rotterdam, the Netherlands; b Azienda Sanitaria Universitaria Integrata del Trentino, Trento, Italy; c Department of Physiotherapy and Rehabilitation Sciences, SOMT University of Physiotherapy, Amersfoort, the Netherlands; d Department of Rheumatology, Leiden University Medical Center, Leiden and Department of Rheumatology, Zuyderland Medical Center, Heerlen, the Netherlands

**Keywords:** Ankylosing spondylitis, Inflammatory back pain, Diagnostic delay, Case finding, Diagnostic performance

## Abstract

**Background:**

Axial spondyloarthritis (axSpA) is a clinically important but often under-recognized condition, with substantial diagnostic delay. Structured referral strategies have been proposed to facilitate earlier identification of suspected cases; however, their diagnostic accuracy has not been comprehensively compared. We therefore conducted a systematic review to synthesize the diagnostic accuracy of referral strategies for identifying axSpA in patients with chronic back pain.

**Methods:**

We performed a systematic review of observational studies evaluating referral strategies for suspected axSpA. The review protocol was prospectively registered in PROSPERO (CRD42025634153). MEDLINE, Embase, Web of Science, CENTRAL, and CINAHL Plus were searched from inception to December 2025. Study selection, data extraction using the “Checklist for Critical Appraisal and Data Extraction for Systematic Reviews of Prediction Modeling Studies” (CHARMS), risk of bias assessment using the “Prediction model Risk Of Bias Assessment Tool + Artificial Intelligence” (PROBAST+AI), and certainty of evidence rating using an adapted “Grading of Recommendations Assessment, Development and Evaluation” (GRADE) approach were conducted by two independent reviewers.

**Findings:**

Twenty-four studies evaluating 40 referral strategies were included. Risk of bias was high across all strategies, resulting in low to very low certainty of evidence for most performance metrics. Meta-analysis was feasible for nine referral strategies. Statistically significant pooled likelihood ratios were observed for the negative likelihood ratio (LR−) of the ASAS referral criteria (0.18, 95% CI 0.04–0.81), and for both the positive and negative LRs of the Braun 2-step alternative strategy (LR+ 2.02, 95% CI 1.47–2.76; LR− 0.38, 95% CI 0.19–0.75), CaFaSpA ≥1 (LR+ 1.64, 95% CI 1.04–2.59; LR− 0.43, 95% CI 0.21–0.87), and RADAR 2/3 (LR+ 2.76, 95% CI 2.13–3.59; LR− 0.60, 95% CI 0.51–0.71). These results did not meet thresholds (e.g., LR+ >10, LR− <0.10) for strong diagnostic accuracy.

**Interpretation:**

Current referral strategies for axSpA have limited diagnostic impact and are supported by low to very low-certainty evidence. Future research should prioritize the development of new referral strategies, particularly for primary care settings.

**Funding:**

No specific funding was received for this work.

**Research in context**

**Evidence before this study**

Axial spondyloarthritis (axSpA) is associated with substantial diagnostic delay, often exceeding six years. Referral strategies have been developed to support early identification among patients with chronic back pain, but their diagnostic performance has been evaluated inconsistently. We searched MEDLINE (via PubMed) and Embase from inception to September 2024 to identify existing systematic reviews on this topic but did not find any that comprehensively synthesized the diagnostic accuracy of referral strategies for axSpA. Therefore, we conducted this systematic review and meta-analysis to evaluate and critically appraise the diagnostic performance of these referral strategies.

**Added value of this study**

This study provides the first comprehensive synthesis and meta-analysis of referral strategies for axSpA. We show that current strategies have limited diagnostic impact, with none demonstrating strong rule-in or rule-out performance. Even the most promising strategies yield only modest changes in post-test probability. We also identify major methodological limitations, including a high risk of bias, poor reporting of key performance domains, and the fact that few strategies have been developed or evaluated in primary care settings, where they are intended to be used.

**Implications of all the available evidence**

Current referral strategies should be used as support tools rather than standalone decision rules. Reducing diagnostic delay in axSpA will require the development of new, primary care–based referral strategies using rigorous data-driven methods and robust validation. Future research should prioritize well-designed studies with complete reporting and evaluation of clinical utility.

## Introduction

Back pain represents the condition responsible for the highest number of years lived with disability, according to the Global Burden of Disease studies.^1^ Despite its substantial societal and healthcare impact, the etiology of back pain remains unidentified in the majority of cases.^2^^,^^3^ This diagnostic uncertainty often leads to broadly applied management strategies that may be suboptimal for specific, clinically relevant patient subgroups in which an underlying cause can be identified.^2^^,^^4^ Among these, axial spondyloarthritis (axSpA) represents a clinically important but frequently under-recognized inflammatory rheumatic and musculoskeletal disease.^5^ Indeed, a significant challenge in axSpA care is the persistent and substantial diagnostic delay, with meta-analytic evidence suggesting a pooled mean delay of approximately 6.7 years worldwide.^6^ Importantly, it has been estimated that up to one-third of patients with chronic back pain of unknown origin and symptom duration of less than two years that are referred to the rheumatologist may already have axSpA.^7^ This diagnostic delay is clinically relevant, as delayed initiation of effective treatment may contribute to ongoing disease activity and worse long-term outcomes.^8^^,^^9^

Multiple factors have been implicated in axSpA delayed diagnosis, including limited awareness among patients and healthcare professionals, time constraints in primary care, persistent misconceptions about the disease (such as the belief that it primarily affects men), limited diagnostic accuracy of individual laboratory or imaging tests, and unclear referral pathways.^10^^,^^11^ Importantly, axSpA represents an uncommon cause for a very common symptom (i.e., chronic back pain), further complicating timely recognition.^10^ To address these challenges, structured referral strategies have been proposed to support early identification of patients with suspected axSpA.^10^^,^^12^^,^^13^ These strategies typically employ algorithms combining symptoms, laboratory findings, imaging results, and extra-musculoskeletal features to guide referral to rheumatology.^12^ Their overarching aim is to balance sensitivity and specificity, maximizing case detection while avoiding an excessive number of inappropriate referrals that could overwhelm specialist services.^13^ One example of referral strategy is the “Assessment of SpondyloArthritis International Society” (ASAS) referral criteria, which consider patients as referral-positive when at least one of several predefined features is present in patients with chronic back pain with onset before 45 years of age (i.e., inflammatory back pain, good response to non-steroidal anti-inflammatory drugs (NSAIDs), family history of SpA, extra-musculoskeletal manifestations, HLA-B27 positivity, elevated acute-phase reactants, or sacroiliitis on imaging).^14^

Despite the clinical importance of early referral and the widespread use of referral strategies, to our knowledge, no systematic review has comprehensively compared the diagnostic accuracy of existing referral strategies for patients with chronic back pain and suspected axSpA. Therefore, the objective of this systematic literature review was to synthesize and critically appraise the evidence on the diagnostic accuracy of referral strategies for identifying axSpA in patients with chronic back pain, with the broader aim of informing clinical decision-making regarding the most appropriate referral approach and identifying gaps in the current evidence base.

## Methods

The protocol of this systematic review was prospectively registered in the PROSPERO database (CRD42025634153), and this manuscript is reported in accordance with the “Transparent Reporting of a multivariable prediction model for Individual Prognosis Or Diagnosis–Systematic Review and Meta-Analysis” (TRIPOD-SRMA) statement.^15^

### Search strategy and study selection

A systematic literature search was conducted in MEDLINE, Embase, Web of Science, Cochrane Central Register of Controlled Trials (CENTRAL), and CINAHL Plus. Duplicate records were removed using EndNote.^16^ The initial search was performed on 20 December 2024 and was updated on 5 December 2025. In addition, backward and forward citation tracking of included studies was conducted in January 2026 (Google Scholar) to identify further potentially relevant studies. The search strategy was developed and implemented in collaboration with an experienced information specialist (Supplementary Material S1).

Study selection was conducted in duplicate, with two reviewers (D.F. and R.W.) independently screening all records. Covidence was used to screen titles and abstracts first, followed by full-text assessment of potentially eligible studies. Disagreements at any stage of the selection process were resolved through discussion or, when necessary, by consultation with a third reviewer (A.C.).

### Eligibility criteria

We included observational studies evaluating the diagnostic accuracy of referral strategies designed to identify patients with suspected axSpA among those with chronic back pain, with the target disease defined either by a rheumatologist's clinical judgment or by established classification criteria. Eligible studies were required to report measures of diagnostic accuracy, including classification, discrimination, and calibration measures, or to provide sufficient data to allow their derivation (e.g., from a 2 × 2 contingency table).

We considered eligible referral strategies applied in any healthcare setting, including primary, secondary, tertiary, and community settings. Referral strategies were defined as clinical tools intended to support the identification of patients who should be referred for specialist rheumatological assessment, rather than to establish a definitive diagnosis. Accordingly, studies in which the evaluated referral strategy was explicitly designed to confirm or rule out axSpA were excluded, as they fell outside the scope of screening.

### Data extraction

After a piloting phase of three studies, data extraction was conducted in accordance with the “Checklist for Critical Appraisal and Data Extraction for Systematic Reviews of Prediction Modeling Studies” (CHARMS).^17^ Extracted variables were, when appropriate, adapted to align with the current nomenclature recommended in the literature. For example, when reported, the term “extra-articular manifestations” was harmonized to “extra-musculoskeletal manifestations,” and the term “ankylosing spondylitis (AS)” was replaced with “radiographic axial spondyloarthritis (r-axSpA)” to ensure consistency with contemporary terminology.^18^^,^^19^

The diagnostic accuracy of referral strategies was assessed by extracting metrics of classification (e.g., sensitivity and specificity, likelihood ratios), discrimination (e.g., area under the receiver operating characteristic curve), calibration (e.g., calibration intercept and slope), and clinical utility (e.g., decision curve analysis).^20^ When likelihood ratios (LRs) were reported, they were directly extracted; when not explicitly reported, they were calculated from sensitivity and specificity using standard formulas (i.e., LR+ = sensitivity/[1 − specificity]; LR− = [1 − sensitivity]/specificity), as LRs are more clinically informative measures that directly support the estimation of post-test probabilities and facilitate interpretation of results.^21^ Performance metrics were categorized as apparent, internally validated, or externally validated.^22^ Metrics were considered apparent when derived from evaluation within the same dataset used to develop the referral strategy^22^; the same metrics were classified as internally validated when performance was assessed in the development dataset but adjusted for overfitting using appropriate statistical techniques, such as bootstrapping or cross-validation^22^; metrics were considered externally validated when performance was evaluated in an independent dataset distinct from the one used for model development.^22^ When data required to reconstruct 2 × 2 contingency tables were unavailable, the corresponding authors were contacted to request additional information. As a secondary outcome, data on costs associated with referral strategies were also extracted, including expenses related to unnecessary rheumatology consultations for patients referred but not diagnosed with axSpA. Data extraction was performed in duplicate, with two reviewers (D.F. and R.W.) independently extracting data from all included studies. Discrepancies were resolved through discussion or, when necessary, by consultation with a third reviewer (A.C.).

### Risk of bias and applicability assessment

Risk of bias and applicability concerns were assessed using the “Prediction model Risk Of Bias Assessment Tool + Artificial Intelligence” (PROBAST+AI).^23^ In accordance with PROBAST+AI guidance, risk of bias was assessed separately for development studies and for individual performance metrics. The risk of bias in the predictors domain was evaluated from the perspective of a non-rheumatologist, as referral strategies are intended for use in non-specialist settings. Accordingly, when predictors were insufficiently described or operationalized, this was taken into account in the risk of bias judgment, even if their meaning might be self-evident within rheumatological practice. The risk of bias in the outcome domain was rated as high when the diagnosis of axSpA was not established by a rheumatologist or was evaluated by adopting classification criteria. An overall high risk of bias judgment was assigned whenever at least one domain was rated as high risk of bias.^23^ Two reviewers conducted the assessments independently (D.F. and R.W.), and disagreements were resolved through discussion or consultation with a third reviewer (A.C.).

### Data analysis and assessment of the certainty of the evidence

Meta-analyses were performed when at least two validated performance metrics (either internally or externally validated) were available for the same referral strategy. Pooling was restricted to classification measures (i.e., sensitivity and specificity). Discrimination and calibration measures were not meta-analyzed because no studies evaluated multiple referral strategies reporting these performance domains, precluding meaningful quantitative synthesis. Given the limited number of studies available for each referral strategy, univariate random-effects meta-analyses were conducted for sensitivity and specificity for descriptive purposes only. A bivariate meta-analytic approach was not pursued because the small number of studies (4 or fewer) would have yielded unstable estimates.^24^ Consequently, pooled sensitivity and specificity estimates should be interpreted as descriptive summaries of the available evidence rather than precise estimates of true diagnostic performance. To address zero or near-zero cells in sensitivity or specificity, we applied a continuity correction of 0.5 to ensure model convergence and stability. Also, leave-one-out sensitivity analyses were additionally performed for all meta-analyses including more than two studies to assess the influence of individual studies on the pooled estimates. Meta-analyses were performed using the Meta-Disc 2.0 software.^25^

We did not assess the risk of small-study effects because the limited number of studies included in the meta-analyses would have made the interpretation of funnel plots unreliable. Also, prediction intervals were not calculated as the very small number of studies per referral strategy (a maximum of four) would have produced unstable and unreliable estimates, thereby limiting their meaningful interpretation. Statistical heterogeneity was quantified using the I^2^ statistic. However, I^2^ represents a relative rather than an absolute measure of heterogeneity and is influenced by both within-study precision and the number of included studies; therefore, it should be interpreted with caution.^26^

For interpretative purposes, LR+ values greater than 10 and LR− values lower than 0.1 were considered indicative of strong rule-in and rule-out performance, respectively.^27^ Also, LRs were considered statistically significant when the 95% confidence intervals did not include the null value of 1, indicating a statistically meaningful change in post-test probability. Positive and negative predictive values were not calculated when not reported, as they are directly influenced by the prevalence of axSpA in the study population and therefore lack transportability across different clinical settings.^28^

The certainty of the evidence was assessed using adapted “Grading of Recommendations Assessment, Development and Evaluation” GRADE approaches for prognostic factors, calibration of prognostic models, and discrimination performance of prognostic models.^29^, ^30^, ^31^ Supplementary Material S2 provides a detailed description of how the GRADE approach was operationalized and applied in this review.

### Protocol amendments

Two methodological amendments were made after protocol registration and before the corresponding analyses were conducted. First, the protocol originally planned to distinguish between diagnostic test accuracy studies and diagnostic prediction models, with separate data extraction procedures and risk-of-bias assessments. However, after further methodological consideration, all referral strategies were conceptualized as prediction diagnostic models because they combine multiple predictors to estimate the probability of axSpA and support referral decisions. Consequently, a unified framework for data extraction, risk-of-bias assessment, and evidence synthesis was applied across all referral strategies. Second, although the protocol specified that certainty of evidence would be assessed using GRADE, the operationalization of this assessment was not described in detail. Prior to conducting the certainty assessments, we therefore developed a more detailed framework for applying an adapted GRADE approach to prediction model performance measures (Supplementary Material S2). This amendment was considered necessary because no definitive GRADE guidance currently exists for evaluating the certainty of evidence in prediction model research.

### Role of the funding source

No specific funding was received for this work.

## Results

**Fig. 1:**
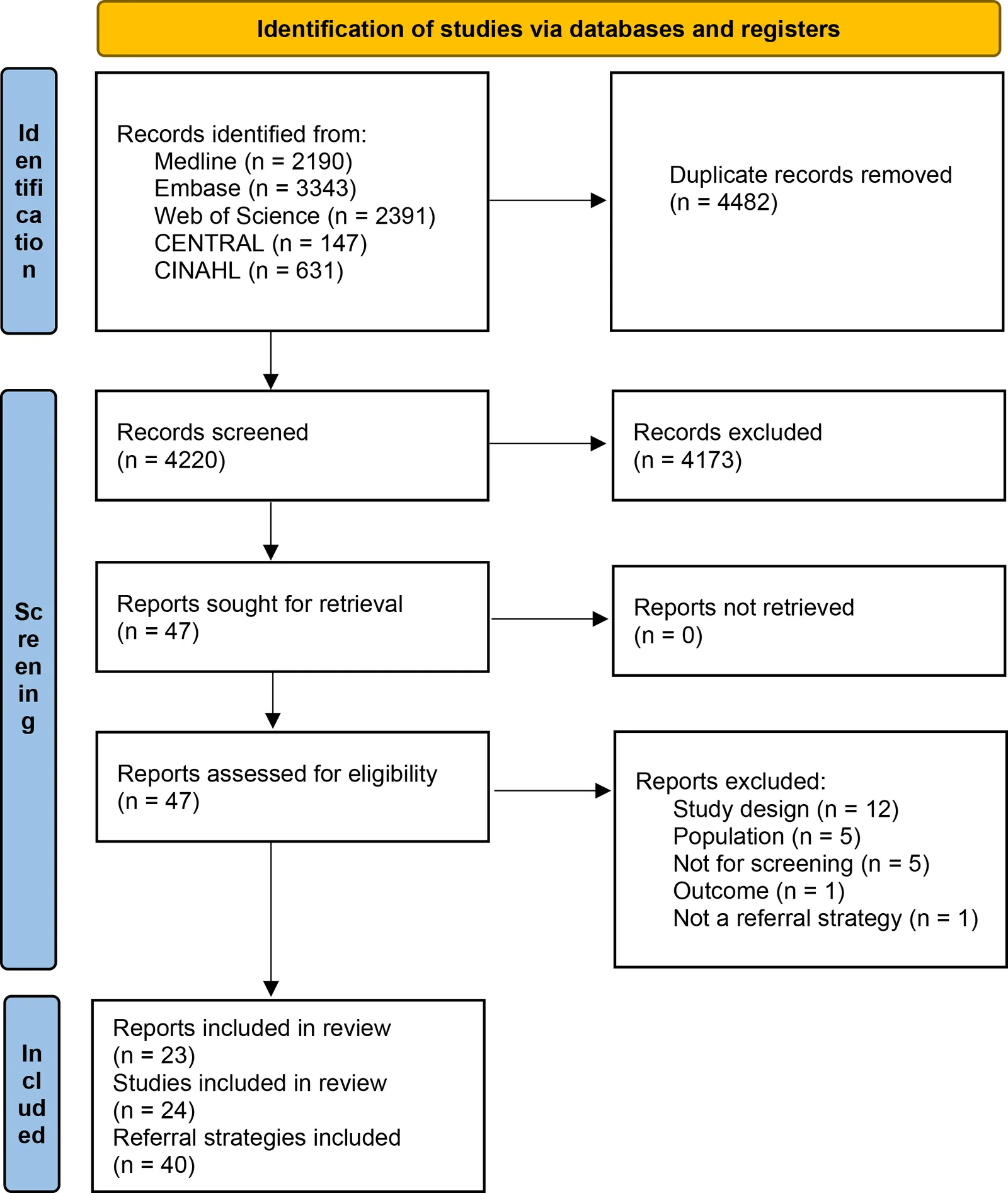
PRISMA flow diagram of study identification, screening, and inclusion.

A total of 8702 records were identified from database searching, and 4482 duplicates were removed. Of the remaining 4220 records screened, 47 full-text articles were assessed for eligibility, resulting in 23 reports included in the review (Fig. 1). Backward and forward citation tracking did not identify any additional eligible studies. Supplementary Material S3 provides a list of all studies excluded after full-text assessment, together with the corresponding reasons for exclusion.

### Characteristics of the included studies

Across the 23 included reports, 24 distinct studies were identified (one report by Maksymowych et al. included two separate study populations^32^), evaluating a total of 40 referral strategies. The median number of participants per study was 317 (range 28–3877; interquartile range 201). On average, 32.2% (SD 13.6) of participants of the eligible studies were diagnosed with axSpA. The mean age across studies was 37.3 years (SD 5.86), and 42.3% (SD 11.1) of participants were male. Table 1 lists all referral strategies, their components, and the corresponding studies that evaluated their performance. Characteristics of the included studies are provided in Supplementary Material S4. Studies were conducted across a range of healthcare settings. Twelve studies were conducted in primary care–based referral settings, six in secondary care rheumatology settings, and two in mixed referral settings (including patients with chronic back pain who self-referred via an online tool or were referred by physicians to specialized rheumatology centers). In addition, two studies used primary care recruitment settings based on general practitioner databases, one study was conducted in a community-based self-referral setting, and one study used a population-based screening approach.

**Table 1:** Referral strategies characteristics (23 studies on 40 different referral strategies).

Referral strategy	Clinical parameters	Laboratory or imaging parameters	Refer if	First author (year)	Performance measure	Method used for the diagnosis of axSpA	n	% axSpA	Age at baseline mean (SD) [range]	Gender (M)
ASAS referral criteria	• IBP defined by any set of criteria, preferably ASAS • Good response to NSAIDs • Family history of SpA • Peripheral manifestations • Extra-musculoskeletal manifestations	• HLA-B27 • Elevated acute-phase reactants • Sacroiliitis (on pelvic radiographs or MRI sacroiliaic joints)	Patients aged ≤45 years with chronic (>3 months) LBP and ≥1/8 features	Abawi (2017)	EV	Rheumatologist's diagnosis and ASAS classification criteria	261	40.9%	31.0 (8.8)	33%
				Maksymowych (2025) (SASPIC-1)	EV	Rheumatologist's diagnosis	212	51.9%	NI	51%
				Maksymowych (2025) (SASPIC-2)	EV	Rheumatologist's diagnosis	151	36.4%	NI	50%
ASAS referral criteria by Sepriano	• IBP defined by any set of criteria, preferably ASAS • Good response to NSAIDs • Family history of SpA • Extra-musculoskeletal manifestations	• HLA-B27 • Elevated acute-phase reactants • Sacroiliitis (on X-rays or MRI)	≥1/7	Sepriano (2019)	AP	Rheumatologists' diagnosis (consensus across three clinicians)	3877	2.4%	56.6 (15.4)	32.2%
ASAS referral criteria by Ziade	• IBP defined by any set of criteria, preferably ASAS • Good response to NSAIDs • Family history of SpA • Peripheral manifestations • Extra-musculoskeletal manifestations		≥1/5	Ziade (2023)	AP	Rheumatologist's diagnosis	471	52.9%	34.8 (7.1)	46.1%
A-tool	• IBP • Enthesitis • peripheral arthritis • psoriasis • IBD • Uveitis • family history of SpA		≥3/8	Danve (2025)	AP	Rheumatologist's diagnosis	86	33.7%	43.7 (10.1)	33%
AWARE ≥1/5	• Improvement with exercise, not with rest • Waking up in the second half of the night • Alternating buttock pain • Good response to NSAIDs (<48 h) • Age ≤35 years		≥1/5	Braun (2019)	AP	Rheumatologist's diagnosis	343	41.1%	NI	NI
AWARE ≥2/5–Braun IBP	• Improvement with exercise, not with rest • Waking up in the second half of the night • Alternating buttock pain • Good response to NSAIDs (<48 h) • Age ≤35 years		≥2/5	Abawi (2017)	EV	Rheumatologist's diagnosis and ASAS classification criteria	261	40.9%	31.0 (8.8)	33%
				Braun (2011)	AP	Rheumatologist's diagnosis	322	35.1%	36.0 (7.9)	49.4%
				Braun (2019)	EV	Rheumatologist's diagnosis	343	41.1%	NI	NI
				Sepriano (2019)	EV	Rheumatologists' diagnosis (consensus across three clinicians)	3877	2.4%	56.6 (15.4)	32.2%
				Ziade (2023)	EV	Rheumatologist's diagnosis	471	52.9%	34.8 (7.1)	46.1%
AWARE ≥3/5	• Improvement with exercise, not with rest • Waking up in the second half of the night • Alternating buttock pain • Good response to NSAIDs (<48 h) • Age ≤35 years		≥3/5	Braun (2019)	AP	Rheumatologist's diagnosis	343	41.1%	NI	NI
Baraliakos combination I	• Good response to NSAIDs • Morning stiffness >30 min	• HLA-B27 • Elevated CRP	≥2/3 or 1 plus HLAB27+	Baraliakos (2020)	AP	Rheumatologist's diagnosis	326	14.1%	36.0 (10.3)	50.6%
Baraliakos combination II	• Inflammatory bowel disease • Family history	• HLA-B27 • Elevated CRP	≥2/3 or 1 plus HLAB27+	Baraliakos (2020)	AP	Rheumatologist's diagnosis	326	14.1%	36.0 (10.3)	50.6%
Baraliakos combination III	• Inflammatory bowel disease • Pain in the thoracic spine • Anterior uveitis	• HLA-B27	≥2/3 or 1 plus HLAB27+	Baraliakos (2020)	AP	Rheumatologist's diagnosis	326	14.1%	36.0 (10.3)	50.6%
Brandt I	• IBP defined by: (≥1/3) • Morning stiffness >30 min • Pain at night or in the early morning • Improvement with exercise	• HLA-B27	≥1/2	Abawi (2017)	EV	Rheumatologist's diagnosis and ASAS classification criteria	261	40.9%	31.0 (8.8)	33%
				Sepriano (2019)	EV	Rheumatologists' diagnosis (consensus across three clinicians)	3877	2.4%	56.6 (15.4)	32.2%
				Ziade (2023)	EV	Rheumatologist's diagnosis	471	52.9%	34.8 (7.1)	46.1%
Brandt I plus imaging	• IBP defined by: (≥1/3) • Morning stiffness >30 min • Pain at night or in the early morning • Improvement with exercise	• HLA-B27 • Sacroiliitis (on any imaging)	≥1/3	Brandt (2007)	AP	Rheumatologist's diagnosis	350	45.4%	40 [16–75]	48.6%
				Proft (2020)	EV	Rheumatologist's diagnosis	181	39.2%	37.4 (11.7)	55.8%
Brandt II	• IBP defined by: (≥2/3) • Morning stiffness >30 min • Pain at night or in the early morning • Improvement with exercise	• HLA-B27	≥1/2	Abawi (2017)	EV	Rheumatologist's diagnosis and ASAS classification criteria	261	40.9%	31.0 (8.8)	33%
				Sepriano (2019)	EV	Rheumatologists' diagnosis (consensus across three clinicians)	3877	2.4%	56.6 (15.4)	32.2%
				Ziade (2023)	EV	Rheumatologist's diagnosis	471	52.9%	34.8 (7.1)	46.1%
Brandt III	• IBP defined by: (≥3/3) • Morning stiffness >30 min • Pain at night or in the early morning • Improvement with exercise	• HLA-B27	≥1/2	Abawi (2017)	EV	Rheumatologist's diagnosis and ASAS classification criteria	261	40.9%	31.0 (8.8)	33%
				Sepriano (2019)	EV	Rheumatologists' diagnosis (consensus across three clinicians)	3877	2.4%	56.6 (15.4)	32.2%
				Ziade (2023)	EV	Rheumatologist's diagnosis	471	52.9%	34.8 (7.1)	46.1%
Braun 2-step	• Improvement with exercise, not with rest • Buttock pain (both sides) • Psoriasis	• HLA-B27 (tested only if ≤ 1/3 clinical parameters positive)	≥2/3 or HLAB27+	Abawi (2017)	EV	Rheumatologist's diagnosis	261	40.9%	31.0 (8.8)	33%
				Braun (2013)	AP	Rheumatologist's diagnosis	298	36.9%	axSpA: 34.2 years Not axSpA: 36.6 years	NI
Braun 2-step alt.	• Improvement with exercise, not with rest • Alternating buttock pain • Psoriasis	• HLA-B27 (tested only if ≤ 1/3 clinical parameters positive)	≥2/3 or HLAB27+	Abawi (2017)	EV	Rheumatologist's diagnosis and ASAS classification criteria	261	40.9%	31.0 (8.8)	33%
				Baraliakos (2020)	EV	Rheumatologist's diagnosis	326	14.1%	36.0 (10.3)	50.6%
Braun 2-step modified	• Improvement with exercise, not with rest • Buttock pain (both sides) • Psoriasis		≥2/3	Sepriano (2019)	AP	Rheumatologists' diagnosis (consensus across three clinicians)	3877	2.4%	56.6 (15.4)	32.2%
CaFaSpA	• IBP defined by the ASAS criteria (≥4/5) (1 point): • Age at onset <40 years • Insidious onset • Improvement with exercise • No improvement with rest • Pain at night (with improvement on getting up) • Positive response to NSAID (1 point) • Family history of SpA (1 point) • More than 5 years of back pain duration (0.5 point)		≥1.5	Van Hoeven (2014)	IV	ASAS classification criteria	364	24.8%	axSpA: 38.6 (6.6) not axSpA: 36.2 (6.9)	axSpA: 38% not axSpA: 44%
				Van Hoeven (2015)	EV	ASAS classification criteria	579	16.4%	35.9 (7.0)	41%
CaFaSpA ≥1	• IBP defined by the ASAS criteria (≥4/5 of the following): • Age at onset <40 years • Insidious onset • Improvement with exercise • No improvement with rest • Pain at night (with improvement on getting up) • Good response to NSAIDs • Family history of SpA		≥1/3	Abawi (2017)	EV	Rheumatologist's diagnosis and ASAS classification criteria	261	40.9%	31.0 (8.8)	33%
				Sepriano (2019)	EV	Rheumatologists' diagnosis (consensus across three clinicians)	3877	2.4%	56.6 (15.4)	32.2%
				Ziade (2023)	EV	Rheumatologist's diagnosis	471	52.9%	34.8 (7.1)	46.1%
CaFaSpA ≥1.5	• IBP defined by the ASAS criteria (≥4/5 of the following): • Age at onset <40 years • Insidious onset • Improvement with exercise • No improvement with rest • Pain at night (with improvement on getting up) • Good response to NSAIDs • Family history of SpA		≥1.5/3	Sepriano (2019)	AP	Rheumatologists' diagnosis (consensus across three clinicians)	3877	2.4%	56.6 (15.4)	32.2%
CaFaSpA ≥2	• IBP defined by the ASAS criteria (≥4/5 of the following): • Age at onset <40 years • Insidious onset • Improvement with exercise • No improvement with rest • Pain at night (with improvement on getting up) • Good response to NSAIDs • Family history of SpA		≥2/3	Abawi (2017)	EV	Rheumatologist's diagnosis and ASAS classification criteria	261	40.9%	31.0 (8.8)	33%
				Sepriano (2019)	AP	Rheumatologists' diagnosis (consensus across three clinicians)	3877	2.4%	56.6 (15.4)	32.2%
Calin's criteria for IBP	• Persistent back pain for ≥3 months • Age of onset <40 years • Insidious onset of back pain • Back pain relieved by exercise • Back stiffness especially in the morning		≥4/5	Abawi (2017)	EV	Rheumatologist's diagnosis and ASAS classification criteria	261	40.9%	31.0 (8.8)	33%
				Hermann (2009)	AP	Rheumatologist's diagnosis	92	32.6%	axSpA: median 32.8 [27.3–38.3] not axSpA: median 36.5 [32.8–41.0]	axSpA: 50% not axSpA: 37.1%
				Sepriano (2019)	EV	Rheumatologists' diagnosis (consensus across three clinicians)	3877	2.4%	56.6 (15.4)	32.2%
				Ziade (2023)	EV	Rheumatologist's diagnosis	471	52.9%	34.8 (7.1)	46.1%
EpiReumaPt	• Previous SpA/PsA diagnosis • IBP (≥3/8): • Onset LBP <40 years • Insidious onset • Improvement with exercise • No improvement with rest • Occurs at night and improves in the morning • Awakening by back pain in the second half of the night • Morning stiffness ≥30 min • Alternating buttock pain • LBP ≥3 months, age <45 years and ≥1/6 SpA features: • Family history of SpA • Previous diagnosis of uveitis by an ophthalmologist • Previous diagnosis of psoriasis by a physician • Previous diagnosis of inflammatory bowel disease by a physician • Good response to NSAIDs • Informed by a physician to be HLA-B27 positive • Dactylitis • Heel enthesitis		≥1/5	Sepriano (2019)	AP	Rheumatologists' diagnosis (consensus across three clinicians)	3877	2.4%	56.6 (15.4)	32.2%
Hamilton screening tool	• Male sex • Age of onset <33 years • No pain radiation • Pain reduction as day goes on • Pain increases with rest • History of iritis		≥3/6	Hamilton (2013)	AP	Modified New York criteria	190	40.0%	axSpA: 46.9 (95% CI: 44.7–49.1) Sacroiliitis: 40.4 (95% CI: 37.0–43.7) Mechanical back pain: 45.9 (95% CI: 43.8–48.0)	axSpA: 42.5% sacroiliitis: 61% mechanical back pain: 31%
				Xiang (2022)	EV	Rheumatologist's diagnosis	1418	3.2%	54.0 (14.1)	27%
Hamilton screening tool (Xiang version)	• IBP defined by the ASAS criteria (≥3/5 of the following): • Age at onset <40 years • Insidious onset • Improvement with exercise • No improvement with rest • Pain at night (with improvement on getting up) • Arthritis • Enthesitis • Uveitis • Dactylitis • Psoriasis • Crohn's disease/Colitis • Good response to NSAIDs • Family history of axSpA		One of the following entry criteria: LBP ≥3 months and age ≤45 years, or No LBP and arthritis, enthesitis or dactylitis Plus ≥1/9 axSpA features	Xiang (2022)	AP	Rheumatologist's diagnosis	1418	3.2%	54.0 (14.1)	27%
Hermann model	• Spontaneous awakening night pain: OR 10.237 (2.698–35.627) • Provocative pain of SIJs: OR 8.855 (2.201–35.627) • Calin's criteria of IBP: OR 6.690 (1.044–42.856) • Cervical spine sagittal movement (cm): OR 3.239 (1.591–6.595) • Neck pain: OR 0.084 (0.015–0.469)		NI	Herman (2009)	AP	Rheumatologist's diagnosis	92	32.6%	axSpA: median 32.8 [27.3–38.3] not axSpA: median 36.5 [32.8–41.0]	axSpA: 50% not axSpA: 37.1%
MASTER strategy 1	• IBP defined by (≥1/3 of the following): • Morning stiffness >30 min in lower spine • Improvement with exercise, not with rest • Awakening by back pain at night, with improvement by exercise	• HLA-B27 • Sacroiliitis on imaging	≥1/3	Poddubnyy (2011)	AP	Rheumatologist's diagnosis	318	41.8%	38.1 (11.3)	53.1%
MASTER strategy 2	• IBP defined by (≥1/3 of the following): • Morning stiffness >30 min • Improvement with exercise not with rest • Pain at night • Good response to NSAIDs • Family history of Ankylosing Spondylitis	• HLA-B27 • Sacroiliitis on imaging	≥2/5	Poddubnyy (2011)	AP	Rheumatologist's diagnosis	242	36.8%	39.5 (10.9)	55.4%
				Ziade (2023)	EV	Rheumatologist's diagnosis	471	52.9%	34.8 (7.1)	46.1%
MASTER without imaging	• IBP defined by (≥1/3 of the following): • Morning stiffness >30 min • Improvement with exercise not with rest • Pain at night • Good response to NSAIDs • Family history of Ankylosing Spondylitis	• HLA-B27	≥2/4	Abawi (2017)	EV	Rheumatologist's diagnosis and ASAS classification criteria	261	40.9%	31.0 (8.8)	33%
MASTER modified	• IBP defined by (≥1/3 of the following): • Morning stiffness >30 min • Improvement with exercise not with rest • Pain at night • Good response to NSAIDs • Family history of r-axSpA		≥2/3	Sepriano (2019)	AP	Rheumatologists' diagnosis (consensus across three clinicians)	3877	2.4%	56.6 (15.4)	32.2%
OptiRef	• Slow onset back pain, no trauma • Back stiffness in the morning >30 min • Improvement with movement or exercise, not with rest • BP second half of the night • Have or had alternating buttock pain • Good response to NSAIDs • Joint pain with swelling and/or tendon inflammation • Psoriasis • Inflammatory bowel disease • Have or had uveitis • Family history of Ankylosing Spondylitis/Psoriasis/Inflammatory bowel disease	• HLA-B27 • Elevated CRP or ESR, not explained by infection	Back pain ≥3 months and age at pain onset <45 years, plus ≥3/13 criteria	Proft (2020)	AP	Rheumatologist's diagnosis	180	19.4%	36.6 (9.2)	42.8%
Passalent screening tool	• Back pain for >3 months • Age of onset <50 years • Morning stiffness >30 min • Nocturnal symptoms occurring in the second half of the night • Improvement of back pain with activity and not with rest		≥3/5	Passalent (2022)	AP	Rheumatologist's diagnosis	405	15.5%	37 (IQR 29–44)	44.9%
RADAR strategy 1	• IBP (not defined)	• HLA-B27 • Sacroiliitis on imaging	≥1/3	Juanola (2013)	EV	Rheumatologist's diagnosis	28	28.6%	35.3 (9.1)	46%
				Sieper (2012)	AP	Rheumatologist's diagnosis	504	34.9%	36.5 (9.93)	47%
RADAR strategy 2	• IBP defined by any set of criteria • Good response to NSAIDs • Family history of SpA • Extra-musculoskeletal manifestations	• HLA-B27 • Sacroiliitis on imaging	≥2/6	Juanola (2013)	EV	Rheumatologist's diagnosis	59	25.4%	37.5 (8.5)	43%
				Sieper (2012)	AP	Rheumatologist's diagnosis	568	38.9%	37.6 (11.73)	54.6%
				Ziade (2023)	EV	Rheumatologist's diagnosis	471	52.9%	34.8 (7.1)	46.1%
RADAR without imaging	• IBP defined by any set of criteria • Good response to NSAIDs • Family history of SpA • Extra-musculoskeletal manifestations	• HLA-B27	≥2/5	Abawi (2017)	EV	Rheumatologist's diagnosis and ASAS classification criteria	261	40.9%	31.0 (8.8)	33%
RADAR 2/3	• IBP defined by any set of criteria • Good response to NSAIDs • Extra-musculoskeletal manifestations		≥2/3	Abawi (2017)	EV	Rheumatologist's diagnosis and ASAS classification criteria	261	40.9%	31.0 (8.8)	33%
				Ziade (2023)	EV	Rheumatologist's diagnosis	471	52.9%	34.8 (7.1)	46.1%
RADAR by Bentin 1/3	• IBP defined by: (≥2/4) • Morning stiffness >30 min • Pain at night or in the early morning that awakens the patient • Improvement with movement, not with rest • Pain in the thighs, alternating between left and right	• HLA-B27 • Sacroiliitis on imaging	≥1/3	Bentin (2012)	AP	Rheumatologist's diagnosis	73	26.0%	34.0 (9.0)	40%
RADAR by Bentin 2/6	• IBP defined by: (≥2/4) • Morning stiffness >30 min • Pain at night or in the early morning that awakens the patient • Improvement with movement, not with rest • Pain in the thighs, alternating between left and right • Family history of axSpA • Good response to NSAIDs • Extra-musculoskeletal manifestations	• HLA-B27 • Sacroiliitis on imaging	≥2/6	Bentin (2012)	AP	Rheumatologist's diagnosis	65	36.9%	36.0 (11.0)	56.1%
SUSPECT	• IBP defined by the ASAS criteria (≥4/5): • Age at onset <40 years • Insidious onset • Improvement with exercise • No improvement with rest • Pain at night (with improvement on getting up) • SpA features: • Inflammatory back pain • Arthritis • Enthesitis of the heel • Uveitis • Dactylitis • Psoriasis • Inflammatory bowel disease • Good response to NSAIDs • Family history of SpA, Chron disease, or psoriasis	• HLA-B27 • Elevated CRP or ESR	IBP plus • ≥1 SpA feature or • HLA-B27+ or • elevated CRP/ESR	Tant (2017)	AP	Rheumatologist's diagnosis	85	43.5%	axSpA: 34.0 (7.7)	axSpA: 30.5%
Weisman model	• Intercept: - 1.02 • Gender (male): OR 3.45 (1.51–7.91) • Pain location: neck OR 3.49 (1.54–7.49), hip OR 3.54 (1.52–8.23), other regions OR 2.57 (1.07–6.15) • Age at onset (years): OR 0.93 (0.89–0.97) • Pain duration (months): OR 1.00 (1.00–1.01) • Numbness/tingling spread into legs (yes): OR 0.36 (0.16–0.81) • Pain/stiffness due to fall/sprain (yes): OR 0.25 (0.10–0.62) • Impact of exercise on pain/stiffness: decreases OR 0.21 (0.06–0.82), increases OR 0.07 (0.01–0.33) • Impact of daily physical activity on pain/stiffness: decreases OR 8.31 (1.92–36.00), increases OR 2.76 (0.69–11.10) • NSAID use and response to the treatment: relief within 48 h OR 1.39 (0.58–3.33), not relief within 48 h OR 0.12 (0.02–0.81) • Diagnosed with iritis (yes): OR 30.31 (7.26–126.49)		Probability >66.86%	Weisman (2010)	AP	NI	316	32.3%	axSpA: 48.6 (13.5) Controls: 43.9 (12.1)	axSpA: 62% Controls: 32%
				Weisman (2010)	IV	NI	135	31.8%	axSpA: 48.6 (13.5) Controls: 43.9 (12.1)	axSpA: 62% Controls: 32%

axSpA = axial spondyloarthritis, r-axSpA = radiographic axial spondyloarthritis, SpA = spondyloarthritis, PsA = psoriatic arthritis, IBP = inflammatory back pain, LBP = low back pain, NSAIDs = non-steroidal anti-inflammatory drugs, CRP = C-reactive protein, ESR = erythrocyte sedimentation rate, SIJs = sacroiliac joints, MRI = magnetic resonance imaging, OR = odds ratio, CI = confidence interval, NI = not indicated, AP = apparent performance, IV = internally validated performance, EV = externally validated performance.

Detailed characteristics of the development studies are provided in Supplementary Material S5. Among the 40 referral strategies, four (i.e., “CaFaSpA”, “Hamilton screening tool”, “Hermann model”, and “Weisman model”) were developed using data-driven statistical methods. The remaining strategies were constructed based on clinically selected predictors without formal model development procedures.

Characteristics related to the development of the referral strategies are reported in Supplementary Material S6. All included studies reported classification measures. Discrimination measures were available for 17 of the 40 referral strategies, whereas calibration was reported only for the CaFaSpA referral strategy. No studies reported measures of clinical utility or costs associated with the implementation of referral strategies.

Performance measures for each referral strategy with accompanying certainty of evidence are summarized in Supplementary Material S7. Overall, all performance metrics for all referral strategies were rated as very low certainty of evidence, except for the specificity of the “MASTER strategy 2”, rated as low certainty, and the specificity of the “RADAR 2/3” strategy, rated as moderate certainty.

### Risk of bias and applicability concerns

**Fig. 2:**
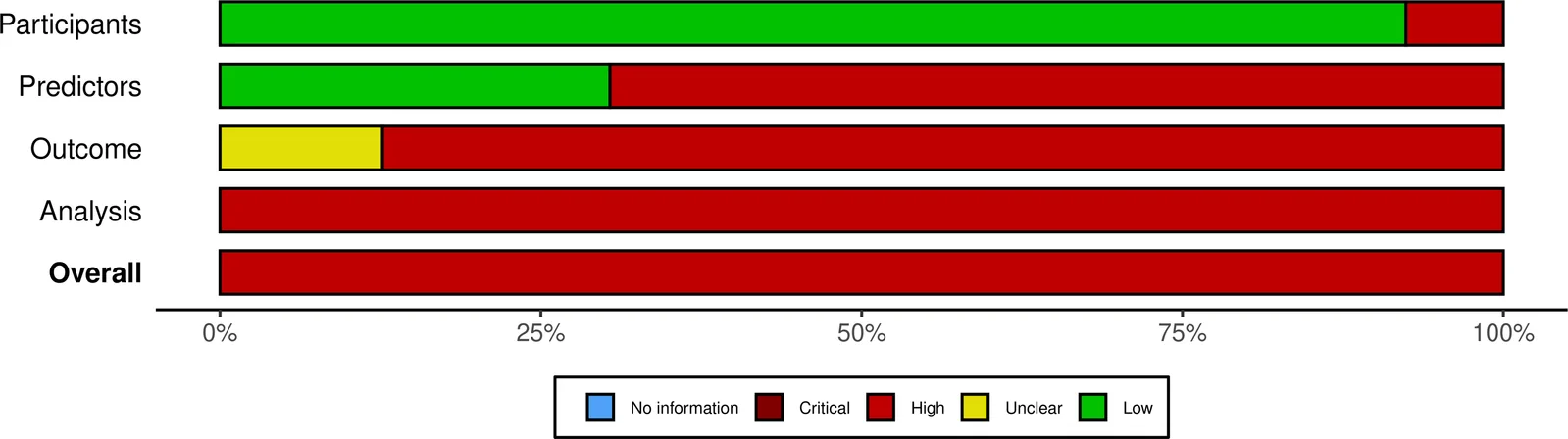
Risk of bias summary across included studies, assessed using PROBAST+AI.

According to the PROBAST+AI, all performance metrics were judged to be at an overall high risk of bias. Although most performance metrics were rated as low risk of bias in the participants domain, substantial concerns emerged in the predictors and outcome domains, where more than 70% of metrics were judged to be at high risk of bias. The analysis domain showed the most pronounced limitations, with all performance metrics rated as high risk of bias (Fig. 2).

**Fig. 3:**
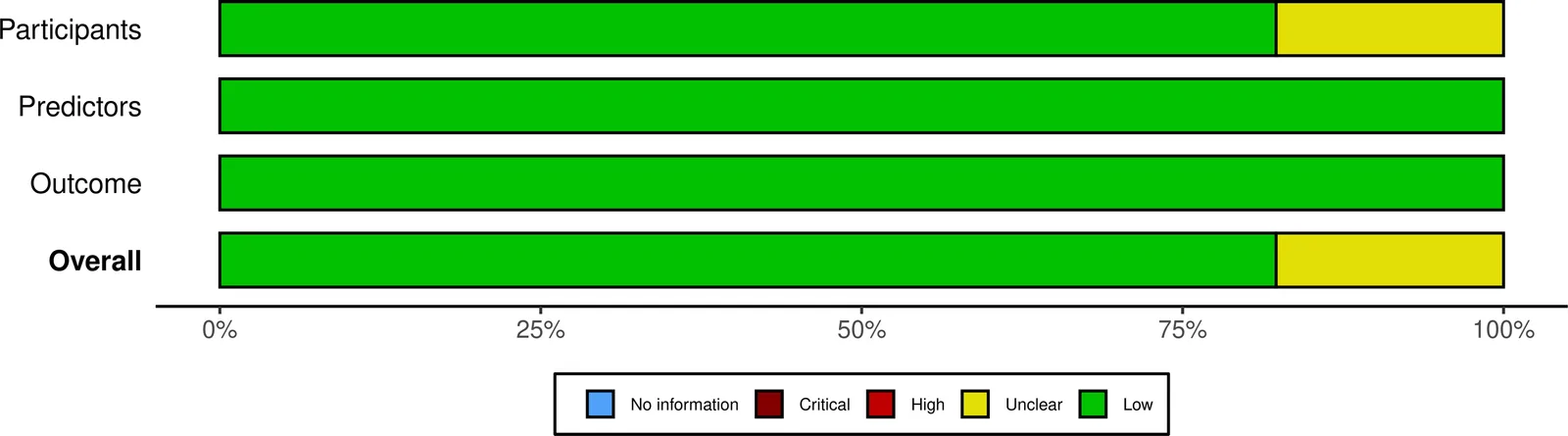
Applicability concerns across included studies, assessed using PROBAST+AI.

In contrast, applicability concerns were generally low. Most performance metrics were rated as having low applicability concerns in the predictors and outcome domains. In the participants domain, the majority were judged as low concern, although a minority were rated as unclear. Overall applicability was considered low across most performance metrics, with only a small proportion judged to have unclear concerns (Fig. 3).

Supplementary Materials S8 and S9 present the detailed risk of bias and applicability assessments for individual performance metrics and referral strategy development studies, respectively.

### Meta—analyses results

Statistical pooling of classification measures was feasible for 9 referral strategies (Table 2). Considering LRs, statistically significant results were observed for the LR− of the “ASAS referral criteria” and for both LR+ and LR− of the “Braun 2-step alt” strategy, “CaFaSpA ≥1”, and “RADAR 2/3”.

**Table 2:** Meta-analyses results.

Referral strategy	Studies	Number of patients	% axSpA	Sensitivity (95% CI)	Specificity (95% CI)	LR+ (95% CI)	LR− (95% CI)	I^2^
ASAS referral criteria	Abawi (2017) Maksymowych (2025) (SASPIC-1) Maksymowych (2025) (SASPIC-2)	624	43.6%	88.9% (63.3%–97.4%)	61.3% (24.3%–88.7%)	2.30 (0.85–6.21)	0.18 (0.04–0.81)	Sensitivity: 91.5% Specificity: 98.2%
AWARE 2/5–Braun IBP	Abawi (2017) Braun (2019) Sepriano (2019) Ziade (2023)	4952	11.9%	92.7% (61.9%–99.0%)	23.8% (4.6%–66.8%)	1.22 (0.76–1.94)	0.30 (0.03–3.30)	Sensitivity: 96.3% Specificity: 99.3%
Brandt I	Abawi (2017) Sepriano (2019) Ziade (2023)	4609	9.7%	95.0% (59.8%–99.6%)	30.9% (7.2%–72.1%)	1.38 (0.79–2.40)	0.16 (0.01–2.41)	Sensitivity: 97.0% Specificity: 99.5%
Brandt II	Abawi (2017) Sepriano (2019) Ziade (2023)	4609	9.7%	82.0% (37.1%–97.2%)	62.0% (22.8%–90.0%)	2.16 (0.70–6.62)	0.29 (0.05–1.75)	Sensitivity: 98.5% Specificity: 99.6%
Brandt III	Abawi (2017) Sepriano (2019) Ziade (2023)	4609	9.7%	46.6% (11.8%–85.2%)	87.1% (52.5%–97.6%)	3.60 (0.56–23.31)	0.61 (0.25–1.52)	Sensitivity: 97.3% Specificity: 99.5%
Braun 2-step alt.	Abawi (2017) Baraliakos (2020)	587	26.1%	76.5% (57.4%–88.7%)	62.1% (52.8%–70.5%)	2.02 (1.47–2.76)	0.38 (0.19–0.75)	Sensitivity: 85.3% Specificity: 85.1%
CaFaSpa 1	Abawi (2017) Sepriano (2019) Ziade (2023)	4609	9.7%	76.9% (60.5%–87.9%)	53.2% (34.2%–71.3%)	1.64 (1.04–2.59)	0.43 (0.21–0.87)	Sensitivity: 92.9% Specificity: 97.9%
Calin's criteria	Abawi (2017) Sepriano (2019) Ziade (2023)	4609	9.7%	70.1% (30.3%–92.7%)	61.4% (21.7%–90.1%)	1.82 (0.55–5.95)	0.49 (0.12–1.90)	Sensitivity: 98.3% Specificity: 99.7%
RADAR 2/3	Abawi (2017) Ziade (2023)	732	48.6%	50.7% (43.0%–58.4%)	81.6% (77.4%–85.2%)	2.76 (2.13–3.59)	0.60 (0.51–0.71)	Sensitivity: 71.7% Specificity: 0%

For the “ASAS referral criteria” (3 studies; very low certainty of evidence), the pooled LR− was 0.18 (95% CI 0.04–0.81), whereas the pooled LR+ was not statistically significant (2.30, 95% CI 0.85–6.21). For the “Braun 2-step alt” strategy (2 studies; very low certainty of evidence), both the pooled LR+ (2.02, 95% CI 1.47–2.76) and LR− (0.38, 95% CI 0.19–0.75) were statistically significant. For the “CaFaSpA ≥1” strategy (3 studies; very low certainty of evidence), the pooled LR+ was 1.64 (95% CI 1.04–2.59) and the pooled LR− was 0.43 (95% CI 0.21–0.87), both reaching statistical significance. For the “RADAR 2/3” strategy (2 studies; very low certainty of evidence), both the pooled LR+ (2.76, 95% CI 2.13–3.59) and LR− (0.60, 95% CI 0.51–0.71) were statistically significant. For all remaining referral strategies included in meta-analyses, pooled LRs were not statistically significant.

Supplementary Material S10 presents the forest plots for all meta-analyses. Supplementary Material S11 presents leave-one-out sensitivity analyses for all meta-analyses including more than two studies. This analysis showed that the pooled estimates varied after exclusion of individual studies, reflecting the small number of studies included in each meta-analysis. Supplementary Material S12 provides the corresponding 2 × 2 contingency tables extracted from all studies contributing to the quantitative syntheses.

### Referral strategies not included in meta-analyses

For the remaining referral strategies, meta-analysis was not feasible due to the absence of multiple validated performance estimates. The majority of these strategies reported only apparent performance measures derived from the development dataset, thereby limiting confidence in their reported diagnostic accuracy.

The “Brandt I plus imaging” strategy was externally validated in one study^33^ (n = 181; 39.2% axSpA; very low certainty of evidence), which reported only a positive predictive value of 39.2% and no additional classification measures.

In a single external validation study by Abawi et al.^34^ (n = 261; 40.9% axSpA; very low certainty of evidence), the “Braun 2-step” strategy showed a sensitivity of 75%, specificity of 59%, LR+ of 1.83, and LR− of 0.42. In the same cohort, “CaFaSpA ≥2” yielded a sensitivity of 51%, specificity of 74%, LR+ of 1.98, and LR− of 0.66. The “MASTER without imaging” strategy demonstrated a sensitivity of 64%, specificity of 76%, LR+ of 2.68, and LR− of 0.47, while “RADAR without imaging” showed a sensitivity of 79%, specificity of 63%, LR+ of 2.12, and LR− of 0.33.

The “Hamilton screening tool” was externally validated by Xiang et al.^35^ (n = 1418; 3.2% axSpA; very low certainty of evidence), reporting a sensitivity of 34.8%, specificity of 93.7%, LR+ of 5.52, and LR− of 0.70. The “MASTER strategy 2” was externally validated by Ziade et al.^36^ (n = 471; 52.9% axSpA; very low certainty of evidence), yielding a sensitivity of 67.1%, specificity of 79.7%, LR+ of 3.3, and LR− of 0.41. The “RADAR strategy 1” was externally validated by Juanola et al.^37^ (n = 28; 28.6% axSpA; very low certainty of evidence), who reported a positive predictive value of 28.5%. The “RADAR strategy 2” was externally validated by Ziade et al.^36^ (n = 471; 52.9% axSpA; very low certainty of evidence), reporting a sensitivity of 75.5%, specificity of 75.2%, LR+ of 3.0, and LR− of 0.33, and by Juanola et al.^37^ (n = 59; 25.4% axSpA; very low certainty of evidence), who reported a positive predictive value of 25.4%. Finally, the “Weisman model” underwent internal validation within the development study^38^ (n = 135; 31.8% axSpA; very low certainty of evidence), reporting a sensitivity of 67.4%, specificity of 94.6%, LR+ of 12.48, and LR− of 0.34.

## Discussion

The objective of this systematic review was to synthesize the evidence on the diagnostic performance of referral strategies for identifying axSpA in patients with chronic back pain, with the broader aim of informing clinical decision-making and identifying gaps in the current evidence base. Overall, none of the included referral strategies showed clinically compelling rule-in or rule-out performance. Although some referral strategies yielded statistically significant LRs, the magnitude of these effects was modest. Indeed, none consistently achieved thresholds traditionally considered indicative of strong diagnostic performance (i.e., LR+ >10 or LR− <0.1).^27^

The interpretation of this review's findings is constrained by several important methodological and contextual limitations. First, most studies were not conducted in primary care settings. Instead, many were performed in secondary care or in selected populations with a relatively high prevalence of axSpA, such as patients already referred to rheumatology services. This limits generalizability to primary care and likely inflates performance metrics due to spectrum effects.^39^ The referral strategies intended for use in primary care should be evaluated in the setting in which they are meant to operate. Second, the overall risk of bias was high across all performance metrics. This resulted in very low certainty of evidence for almost all outcomes. Consequently, these methodological limitations substantially restrict confidence in the observed effects. Third, the large majority of referral strategies were developed using just clinically selected predictors rather than combined with data-driven statistical methods. This represents a missed opportunity to identify the most informative combination of predictors and to optimize the weighting of variables. Data-driven approaches may uncover predictor interactions, nonlinear effects, or unexpected combinations of features that are not captured by opinion-based strategies. Fourth, metrics like discrimination, calibration, and clinical utility were almost entirely unreported, which is a critical omission.^40^ For example, without calibration assessment it is impossible to determine whether predicted risks reflect observed probabilities.^40^ Similarly, the absence of decision curve analysis prevents assessment of whether these strategies provide net clinical benefit in practice.^40^ Fifth, we did not identify any data on costs associated with the implementation of these referral strategies, precluding evaluation of their potential economic impact and cost-effectiveness within healthcare systems. Taken together, these findings indicate that the current evidence base is insufficient to support firm recommendations on the use of any specific referral strategy for axSpA in primary care.

Given the current evidence base, no referral strategy can be recommended as a definitive screening tool in isolation. Based on our meta-analyses, the most promising strategies appear to be the RADAR 2/3 for rule-in (pooled LR+ 2.76) and the ASAS referral criteria for rule-out (pooled LR− 0.18).^14^^,^^41^ However, even these LRs translate into only moderate shifts in post-test probability, particularly in the case of the RADAR 2/3. For example, assuming a pre-test probability of 3% in a patient in primary care, a positive RADAR 2/3 result (LR+ 2.76) would increase the post-test probability to approximately 8%, representing a relatively modest absolute change. Nevertheless, RADAR 2/3 has the practical advantage of relying exclusively on clinical features, whereas the ASAS referral criteria may incorporate laboratory or imaging findings when available. This may enhance feasibility in primary care settings where such investigations are not always readily accessible. Among other strategies with statistically significant LRs in the meta-analyses, CaFaSpA ≥1 appears promising particularly for rule-out (LR− 0.43), and its reliance exclusively on clinical parameters increases its pragmatic appeal.^42^

In head-to-head comparisons, some strategies demonstrated relatively more favorable performance. For example, in the study by Sepriano et al.,^43^ Brandt III achieved an LR+ of 5.1, while the ASAS referral strategy (Sepriano version) and EpiReumaPt yielded LR− values of 0.4.^44^ In the study by Ziade et al., MASTER strategy 2 showed the highest LR+ (i.e., 3.3), whereas the modified ASAS referral strategy achieved an LR− of 0.01; however, this latter accurate estimate was based on apparent performance rather than internal or external validation.^36^ Also, all these estimates originate from single studies with an overall high risk of bias, and the lack of reported confidence intervals further undermines their interpretability.

In practice, referral strategies should be considered structured support tools that enhance clinical suspicion rather than deterministic decision algorithms. Their interpretation must account for the pre-test probability of axSpA in the specific clinical context, which may differ markedly across settings, such as self-referral populations, general practice, or secondary care clinics, where the underlying prevalence of axSpA varies substantially. The choice of referral threshold should depend on the acceptable balance between missed cases and unnecessary referrals, and clinicians should explicitly consider how LRs modify post-test probability when deciding whether to refer. Unfortunately, this probabilistic and context-dependent approach remains inconsistently applied across many clinical conditions, and axSpA is not unique in this regard, reflecting a broader tendency in healthcare to underuse formal clinical reasoning in favor of rigid, dichotomous decision rules.^45^, ^46^, ^47^

Future research should not focus on additional validation of existing referral strategies, as many are constrained by their development context, methodological weaknesses, and biased predictor selection processes. Instead, there is a need to develop new referral strategies specifically designed for primary care settings and evaluated in unselected chronic back pain populations. Such strategies should be developed combining clinical expertise with rigorous data-driven techniques, including appropriate sample size calculations to minimize overfitting, avoidance of automatic variable selection procedures (e.g., backward elimination), incorporation of internal validation (e.g., bootstrapping), and subsequent external validation in independent cohorts.^22^ Importantly, future referral strategies should provide an estimated probability of axSpA rather than a purely dichotomous yes/no referral decision, thereby allowing clinicians to better apply acceptable trade-offs between missed cases and unnecessary referrals.^22^ Also, transparent reporting in accordance with TRIPOD+AI is essential. TRIPOD+AI is a reporting guideline that provides recommendations to ensure complete and transparent reporting of prediction model development and validation studies. This is particularly important given the frequent absence of key performance measures observed in the current literature.^48^^,^^49^ Implementing these methodological standards is challenging in the context of axSpA. The relatively low prevalence of axSpA among patients with chronic back pain in primary care populations makes it difficult to assemble sufficiently large and adequately powered cohorts for robust model development and validation.^50^ Achieving an appropriate number of outcome events to ensure stable estimates may require very large sample sizes. In this regard, the use of electronic health records, large primary care registries, and population-based databases may represent a pragmatic solution. Multicentre collaborations and data-sharing initiatives could further facilitate the accrual of sufficiently large datasets. Careful consideration should also be given to balancing model complexity with clinical feasibility. While advanced modeling techniques may improve performance, referral strategies must remain implementable in busy primary care environments.^40^ Integrating digital decision-support tools into electronic medical record systems may represent a promising strategy to enhance clinical usability and facilitate their implementation in routine practice. Finally, several predictors recur consistently across the referral strategies, including inflammatory back pain features, HLA-B27 positivity, response to NSAIDs, extra-musculoskeletal manifestations, and family history of SpA. Future models should formally quantify the independent contribution and optimal weighting of these predictors, while also evaluating additional potentially promising predictors that have been underexplored or inconsistently incorporated in existing strategies.

This systematic review has several strengths. For example, we used validated methodological tools, including PROBAST+AI, and assessed certainty of evidence using an adapted GRADE framework.

However, the added value of applying GRADE in this context may be questioned. Because there is no definitive GRADE guidance for predictive models, we used an adapted approach. Moreover, given that the risk of bias was already high across most performance metrics, the overall certainty of evidence remained very low for nearly all outcomes, and the GRADE assessment did not materially alter the interpretation of the findings. Nonetheless, it provided a transparent and systematic framework for communicating the certainty of the evidence. Another limitation concerns the interpretative cut-offs used for LRs. The thresholds applied in this review (LR+ >10; LR− <0.1) are relatively stringent.^27^ In rheumatology, lower pragmatic thresholds (e.g., LR+ >4; LR− <0.4) are sometimes considered acceptable.^51^^,^^52^ However, even under these less conservative benchmarks, the overall conclusions would not meaningfully change, as most strategies still demonstrated only moderate shifts in post-test probability. Finally, meta-analyses were conducted using univariate models due to the limited number of studies per referral strategy, and heterogeneity was assessed using I^2^, which is a relative measure and should be interpreted cautiously. These methodological constraints primarily reflect the scarcity of validation studies rather than limitations intrinsic to this review.

Currently available referral strategies for axSpA among patients with chronic back pain demonstrate limited diagnostic impact and are supported by low to very-low certainty of evidence. Among the currently available options, the ASAS referral criteria appear relatively useful for rulling-out axSpA diagnosis, while RADAR 2/3 may be comparatively informative for rule-in, according to our meta-analyses; however, their overall impact on post-test probability remains modest. In practice, referral strategies should be considered structured support tools that enhance clinical suspicion rather than deterministic decision algorithms. Future studies should prioritize the development of new, primary care–based referral strategies using rigorous data-driven methodologies and proper validation, as additional validation of currently available strategies is unlikely to overcome their inherent methodological and contextual limitations.

## Contributors

D.F.: Conceptualization, Methodology, Study Selection, Data Extraction, Verification of the underlying data, Data Analysis, Writing.

R.W.: Methodology, Study Selection, Data Extraction, Verification of the underlying data, Writing.

B.K.: Methodology, Writing.

S.R.: Conceptualization, Methodology, Writing.

A.C.: Conceptualization, Methodology, Study Selection, Data Extraction, Writing.

All authors read and approved the final version of the manuscript.

## Data sharing statement

All data relevant to this study are included in the article and its Supplementary Materials. Extracted data and analytic code are available from the corresponding author upon reasonable request.

## Declaration of interests

DF, BK, and RWW declare no competing interests. AC reports research funding from ZonMw (HGOG and GGG programmes) and the European Union Horizon programme, paid to his institution, and serves as vice-chair of an Osteoarthritis Research Society International (OARSI) initiative to develop classification criteria for spinal osteoarthritis (unpaid). SR reports grants or contracts, consulting fees, and honoraria for lectures or educational activities from AbbVie, Alfasigma, Eli Lilly, Johnson & Johnson, MSD, Novartis, Pfizer, Takeda, and UCB; and support for attending meetings or travel from AbbVie, Eli Lilly, and UCB; she is also a member of the Executive Committee of the Assessment of SpondyloArthritis international Society (ASAS).
