# Supplementary material for: Performance of referral strategies for patients with suspected axial spondyloarthritis: a systematic review with meta-analysis
Source: eClinicalMedicine. 2026 Aug 26;99:104167. doi: 10.1016/j.eclinm.2026.104167 (PMC13543815; doi:10.1016/j.eclinm.2026.104167)
Supplement: Supplementary file 1 [file mmc1.docx]

**SUPPLEMENTARY MATERIAL 1 – SEARCH STRATEGIES**

| **Database searched** | **Platform** | **Years of coverage** | **Records** | **Records after duplicates removed** |
| --- | --- | --- | --- | --- |
| Medline ALL | Ovid | 1946 - Present | 2190 | 2183 |
| Embase | Embase.com | 1971 - Present | 3343 | 1394 |
| Web of Science Core Collection* | Web of Knowledge | 1975 - Present | 2391 | 484 |
| Cochrane Central Register of Controlled Trials | Wiley | 1992 - Present | 147 | 62 |
| CINAHL Plus | EBSCO | 1982 - Present | 631 | 97 |
| **Total** | | | **8702** | **4220** |

*Science Citation Index Expanded (1975-present) ; Social Sciences Citation Index (1975-present) ; Arts & Humanities Citation Index (1975-present) ; Conference Proceedings Citation Index- Science (1990-present) ; Conference Proceedings Citation Index- Social Science & Humanities (1990-present) ; Emerging Sources Citation Index (2005-present) No other database limits were used than those specified in the search strategies

No other database limits were used than those specified in the search strategies

**Medline**

(Axial Spondyloarthritis / OR * Spondylarthritis / OR * Spondylarthropathies / OR ((axial* ADJ3 (spondyloarthr* OR spondylarthr* OR spondyl*-arthr*)) OR ((ankylosing* OR ankylotic* OR ankylosis* OR ankylopoi*) ADJ3 (spondylit* OR spondilit* OR spondylarthr* OR spondyl*-arthr* OR spondyloarthr* OR spine* OR spinal* OR vertebra*)) OR axSpA OR Bechterew* OR bekhterev*).ab,ti,kw. OR (spondyloarthr* OR spondylarthr* OR spondyl*-arthr*).ti.) AND ("Referral and Consultation"/ OR Triage / OR Early Diagnosis/ OR Delayed Diagnosis/ OR Mass Screening/ OR screening test/ OR ((Diagnosis/ OR Observer Variation/) AND (Primary Health Care/ OR General Practice/ OR General Practitioners/ OR Physical Therapists/ OR Osteopathic Physicians/)) OR (referral* OR consultation* OR referring* OR refering* OR triage* OR ((early* OR delay*) ADJ3 (diagnos* OR identif* OR detect* OR recogni*)) OR screen* OR (early* ADJ3 (diagnos* OR identif*)) OR screen* OR ((diagnos*) ADJ6 (primary-care OR primary-health-care OR primary-healthcare OR general-practi* OR physiotherapist* OR manual-therap* OR chiropract*))).ab,ti,kw. OR (primary-care OR primary-health-care OR primary-healthcare OR general-practi* OR physiotherapist* OR manual-therap* OR chiropract*).ti.) NOT (exp animals/ NOT humans/) NOT (Case Reports / OR (case ADJ (report* OR series* OR study OR studies)).ti.)

**Embase**

('axial spondyloarthritis'/exp OR spondylarthritis/mj OR spondyloarthropathy/mj OR ((axial* NEAR/3 (spondyloarthr* OR spondylarthr* OR spondyl*-arthr*)) OR ((ankylosing* OR ankylotic* OR ankylosis* OR ankylopoi*) NEAR/3 (spondylit* OR spondilit* OR spondylarthr* OR spondyl*-arthr* OR spondyloarthr* OR spine* OR spinal* OR vertebra*)) OR axSpA OR Bechterew* OR bekhterev*):ab,ti,kw OR (spondyloarthr* OR spondylarthr* OR spondyl*-arthr*):ti) AND ('patient referral'/exp OR 'patient triage'/de OR 'early diagnosis'/de OR 'delayed diagnosis'/de OR screening/de OR 'screening test'/de OR ((diagnosis/de OR 'observer variation'/de) AND ('primary health care'/exp OR 'general practice'/de OR 'general practitioner'/de OR physiotherapist/de OR 'osteopathic physician'/de OR 'manual therapist'/exp)) OR (referral* OR consultation* OR referring* OR refering* OR triage* OR ((early* OR delay*) NEAR/3 (diagnos* OR identif* OR detect* OR recogni*)) OR screen* OR (early* NEAR/3 (diagnos* OR identif*)) OR screen* OR ((diagnos*) NEAR/6 (primary-care OR primary-health-care OR primary-healthcare OR general-practi* OR physiotherapist* OR manual-therap* OR chiropract*))):Ab,ti,kw OR (primary-care OR primary-health-care OR primary-healthcare OR general-practi* OR physiotherapist* OR manual-therap* OR chiropract*):ti) NOT ([animals]/lim NOT [humans]/lim) NOT [conference abstract]/lim NOT 'clinical trial':it NOT ('case report'/de OR 'case study'/de OR (case NEXT/1 (report* OR series* OR study OR studies)):ti)

**Web of science**

(TS=((axial* NEAR/2 (spondyloarthr* OR spondylarthr* OR spondyl*-arthr*)) OR ((ankylosing* OR ankylotic* OR ankylosis* OR ankylopoi*) NEAR/2 (spondylit* OR spondilit* OR spondylarthr* OR spondyl*-arthr* OR spondyloarthr* OR spine* OR spinal* OR vertebra*)) OR axSpA OR Bechterew* OR bekhterev*) OR TI=(spondyloarthr* OR spondylarthr* OR spondyl*-arthr*)) AND (TS=(referral* OR consultation* OR referring* OR refering* OR triage* OR ((early* OR delay*) NEAR/2 (diagnos* OR identif* OR detect* OR recogni*)) OR screen* OR (early* NEAR/2 (diagnos* OR identif*)) OR screen* OR ((diagnos*) NEAR/5 (primary-care OR primary-health-care OR primary-healthcare OR general-practi* OR physiotherapist* OR manual-therap* OR chiropract*))) OR TI=(primary-care OR primary-health-care OR primary-healthcare OR general-practi* OR physiotherapist* OR manual-therap* OR chiropract*)) NOT TI=((case-report* OR case-series* OR case-study OR case-studies)) NOT DT=(Meeting Abstract OR Meeting Summary) AND LA=(english)

**Cochrane**

(((axial* NEAR/3 (spondyloarthr* OR spondylarthr* OR spondyl* NEXT arthr*)) OR ((ankylosing* OR ankylotic* OR ankylosis* OR ankylopoi*) NEAR/3 (spondylit* OR spondilit* OR spondylarthr* OR spondyl* NEXT arthr* OR spondyloarthr* OR spine* OR spinal* OR vertebra*)) OR axSpA OR Bechterew* OR bekhterev*):ab,ti,kw OR (spondyloarthr* OR spondylarthr* OR spondyl* NEXT arthr*):ti) AND ((referral* OR consultation* OR referring* OR refering* OR triage* OR ((early* OR delay*) NEAR/3 (diagnos* OR identif* OR detect* OR recogni*)) OR screen* OR (early* NEAR/3 (diagnos* OR identif*)) OR screen* OR ((diagnos*) NEAR/6 (primary-care OR primary-health-care OR primary-healthcare OR general-practi* OR physiotherapist* OR manual-therap* OR chiropract*))):Ab,ti,kw OR (primary-care OR primary-health-care OR primary-healthcare OR general-practi* OR physiotherapist* OR manual-therap* OR chiropract*):ti)

("conference abstract":kw OR Trial registry record:pt)

#1 NOT #2

**CINAHL**

(MH Axial Spondyloarthritis OR MM Spondylarthritis OR MM Spondylarthropathies OR TI((axial* N2 (spondyloarthr* OR spondylarthr* OR spondyl*-arthr*)) OR ((ankylosing* OR ankylotic* OR ankylosis* OR ankylopoi*) N2 (spondylit* OR spondilit* OR spondylarthr* OR spondyl*-arthr* OR spondyloarthr* OR spine* OR spinal* OR vertebra*)) OR axSpA OR Bechterew* OR bekhterev*) OR AB((axial* N2 (spondyloarthr* OR spondylarthr* OR spondyl*-arthr*)) OR ((ankylosing* OR ankylotic* OR ankylosis* OR ankylopoi*) N2 (spondylit* OR spondilit* OR spondylarthr* OR spondyl*-arthr* OR spondyloarthr* OR spine* OR spinal* OR vertebra*)) OR axSpA OR Bechterew* OR bekhterev*) OR TI(spondyloarthr* OR spondylarthr* OR spondyl*-arthr*)) AND (MH "Referral and Consultation" OR MH Triage OR MH Early Diagnosis OR MH Diagnosis, Delayed OR ((MH Diagnosis OR MH Observer Bias) AND (MH Primary Health Care OR MH Family Practice OR MH Physicians, Family OR MH Physical Therapists OR MH Osteopathic Physicians)) OR TI(referral* OR consultation* OR referring* OR refering* OR triage* OR ((early* OR delay*) N2 (diagnos* OR identif* OR detect* OR recogni*)) OR screen* OR (early* N2 (diagnos* OR identif*)) OR screen* OR ((diagnos*) N5 (primary-care OR primary-health-care OR primary-healthcare OR general-practi* OR physiotherapist* OR manual-therap* OR chiropract*))) OR AB(referral* OR consultation* OR referring* OR refering* OR triage* OR ((early* OR delay*) N2 (diagnos* OR identif* OR detect* OR recogni*)) OR screen* OR (early* N2 (diagnos* OR identif*)) OR screen* OR ((diagnos*) N5 (primary-care OR primary-health-care OR primary-healthcare OR general-practi* OR physiotherapist* OR manual-therap* OR chiropract*))) OR TI(primary-care OR primary-health-care OR primary-healthcare OR general-practi* OR physiotherapist* OR manual-therap* OR chiropract*)) NOT (MH animals+ NOT MH humans+) NOT (MH Case Studies OR TI(case N1 (report* OR series* OR study OR studies)))
