# Supplementary material for: Performance of referral strategies for patients with suspected axial spondyloarthritis: a systematic review with meta-analysis
Source: eClinicalMedicine. 2026 Aug 26;99:104167. doi: 10.1016/j.eclinm.2026.104167 (PMC13543815; doi:10.1016/j.eclinm.2026.104167)
Supplement: Supplementary file 2 [file mmc10.docx]

**Supplementary material 10 – Forest plots**

ASAS:

AWARE 2/5:

Brandt I:

Brandt II:

Brandt III:

Braun 2-step alt.:

CaFaSpA 1:

Calin’s criteria:

Radar 2/3:
