# Supplementary material for: Performance of referral strategies for patients with suspected axial spondyloarthritis: a systematic review with meta-analysis
Source: eClinicalMedicine. 2026 Aug 26;99:104167. doi: 10.1016/j.eclinm.2026.104167 (PMC13543815; doi:10.1016/j.eclinm.2026.104167)
Supplement: Supplementary file 4 [file mmc2.docx]

**SUPPLEMENTARY MATERIAL 2 – GRADE CRITERIA**

The certainty of the evidence was assessed using adapted GRADE approaches for prognostic factors, calibration of prognostic models, and discrimination performance of prognostic models. Certainty ratings were conducted separately for each diagnostic performance domain: sensitivity, specificity, discrimination measures, and calibration measures. For classification performance, we focused on sensitivity, specificity, and likelihood ratios (LR+ and LR−), as these measures were considered the most informative for clinical decision-making and referral purposes. When referral strategies were reported exclusively using predictive values (positive or negative predictive value), these measures were assessed instead. Each outcome was therefore evaluated independently, acknowledging that certainty may differ across performance metrics for the same referral strategy.

When a referral strategy was reported with both apparent performance estimates and validated performance estimates (either internal or external validation), only validated results were considered for the GRADE assessment. Apparent performance estimates were excluded in such cases, as they are prone to diagnostic performance optimism. When only apparent performance estimates were available, these were included but explicitly accounted for the initial certainty rating. Indeed, validated performance estimates were initially rated as high-certainty evidence, whereas outcomes based solely on apparent performance were initially rated as low-certainty evidence, reflecting the overfitting associated with non-validated results.

The certainty of the evidence was downgraded across the following GRADE domains:

1. Risk of bias was assessed using the PROBAST+AI, and downgraded by one level when more than 50% of the contributing studies were judged at overall moderate or unclear risk of bias, and by two levels when more than 50% of the studies were judged at overall high risk of bias.
2. Inconsistency was downgraded when substantial unexplained variability across studies was observed, defined as high statistical heterogeneity in meta-analyses (I² > 80%) or, when meta-analysis was not feasible, as marked differences in point estimates across studies (≥10% absolute difference for sensitivity, specificity, and AUC, or ≥0.10 for calibration slope and calibration intercept). Inconsistency was not assessed when evidence for a given referral strategy was derived from a single study.
3. Indirectness was evaluated based on overall applicability concerns as assessed with PROBAST+AI; evidence was downgraded by one level when more than 50% of studies were rated as having moderate or unclear concerns regarding applicability, and by two levels when more than 50% of studies were judged as having high overall applicability concerns.
4. Imprecision was downgraded when confidence intervals around pooled or individual point estimates were wide enough to include clinically meaningful differences, defined a priori as ≥10% absolute differences for sensitivity, specificity, and AUC, and ≥0.10 for calibration slope and calibration intercept. When confidence intervals were not reported, imprecision was judged based on the effective sample size; given the binary outcome, this was defined as the smallest number of patients with and without axSpA, with downgrading applied when this effective sample size was considered insufficient to provide stable estimates.
5. Publication bias was considered likely and led to downgrading unless evidence for a referral strategy was supported by multiple validation studies, regardless of whether these were internal or external validations, thereby reducing concerns related to selective reporting and overestimation of performance.

Certainty of the evidence was upgraded when predefined criteria were met, including evidence of moderate or large diagnostic performance (defined a priori as an AUC > 0.70, sensitivity > 0.90, or specificity > 0.70), provided that the entire confidence interval lay above the corresponding threshold, or when an exposure–response gradient was observed across risk strata or referral thresholds.
