# Supplementary material for: Performance of referral strategies for patients with suspected axial spondyloarthritis: a systematic review with meta-analysis
Source: eClinicalMedicine. 2026 Aug 26;99:104167. doi: 10.1016/j.eclinm.2026.104167 (PMC13543815; doi:10.1016/j.eclinm.2026.104167)
Supplement: Supplementary file 5 [file mmc3.docx]

**Supplementary material 3 – Reports excluded after full-text assessment**

| **REFERENCE** | **REASON FOR EXCLUSION** |
| --- | --- |
| Alnaqbi KA, Touma Z, Passalent L, Johnson SR, Tomlinson GA, Carty A, Inman RD. Development, sensibility, and reliability of the Toronto Axial Spondyloarthritis Questionnaire in inflammatory bowel disease. J Rheumatol. 2013 Oct;40(10):1726-35. doi: 10.3899/jrheum.130048. Epub 2013 Sep 1. PMID: 23996291. | Wrong patient population |
| Arndt U, Behrens F, Ziswiler HR, Kaltwasser JP, Möller B. Observational study of a patient and doctor directed pre-referral questionnaire for an early arthritis clinic. Rheumatol Int. 2007 Nov;28(1):21-6. doi: 10.1007/s00296-007-0382-x. Epub 2007 Jun 15. PMID: 17571266. | Wrong patient population |
| Benavent D, Tapia M, Bernabeu D, Muley V, Juárez M, Balsa A, Plasencia C, Navarro-Compán V. Can we identify axial spondyloarthritis among young adults referred to non-rheumatology specialists? Results from the SHERPAS study. Semin Arthritis Rheum. 2025 Oct;74:152780. doi: 10.1016/j.semarthrit.2025.152780. Epub 2025 Jul 7. PMID: 40651091. | Strategy not for screening |
| Biln NK, Bansback N, Black C, Shojania K, Guh D, Harrison MJ. The Patient Self-Administered Inflammatory Arthritis Detection Study. J Rheumatol. 2026 May 1;53(5):529-536. doi: 10.3899/jrheum.2025-0428. PMID: 41326158. | Wrong outcome |
| Braun, A.; Gnann, H.; Saracbasi, E.; Grifka, J.; Kiltz, U.; Schnittker, J.; Letschert, K.; Braun, J.. Identification of axial spondyloarthritis among patients with chronic back pain in primary care-how does determination of HLA B27 influence the performance of clinical assessments of inflammatory back pain? Arthritis and rheumatism 2012;64():S241 2012 | Conference abstract |
| Braun A, Saracbasi E, Grifka J, Schnitker J, Braun J. Identifying patients with axial spondyloarthritis in primary care: how useful are items indicative of inflammatory back pain? Ann Rheum Dis. 2011 Oct;70(10):1782-7. doi: 10.1136/ard.2011.151167. Epub 2011 Aug 5. PMID: 21821621; PMCID: PMC3171105. | Strategy not for screening |
| Calin A, Porta J, Fries JF, Schurman DJ. Clinical history as a screening test for ankylosing spondylitis. JAMA. 1977 Jun 13;237(24):2613-4. PMID: 140252. | Wrong study design |
| Deodhar A, Rozycki M, Garges C, Shukla O, Arndt T, Grabowsky T, Park Y. Use of machine learning techniques in the development and refinement of a predictive model for early diagnosis of ankylosing spondylitis. Clin Rheumatol. 2020 Apr;39(4):975-982. doi: 10.1007/s10067-019-04553-x. Epub 2019 May 1. PMID: 31044386. | Strategy not for screening |
| Duruöz MT, Şanal Toprak C, Ulutatar F. Validation of the Toronto Psoriatic Arthritis Screen II (TOPAS II) questionnaire in a Turkish population. Rheumatol Int. 2018 Feb;38(2):255-259. doi: 10.1007/s00296-017-3871-6. Epub 2017 Nov 7. PMID: 29116441. | Wrong patient population |
| Érdes ShF, Bochkova AG, Dubinina TV, Lapshina SA, Miasoutova LI, Rumiantseva OA, Salikhov IG. [The working classification and early diagnostics of ankylosing spondylitis]. Klin Med (Mosk). 2014;92(6):5-11. Russian. PMID: 25799824. | Wrong study design |
| Fonseca JE, Pereira da Silva JA, Bernardes M, Cernadas R, Canas da Silva J, Costa L, Videira T, C Miranda L, Barcelos A, Laires PA, Dezerto R, D Pereira M, Vidal F, Cunha E, Morais A, Martins C, Mesquita R. Effectiveness of a Referral Program for rheumatoid arthritis and axial spondyloarthritis Diagnosis at Primary Care Centers in Portugal - SIARA STUDY. Acta Reumatol Port. 2018 Jan-Mar;43(1):40-51. English. PMID: 29506017. | Wrong study design |
| Hellamand P, van de Sande MGH, van Bentum RE, Verbraak FD, Twisk JWR, van der Horst Bruinsma I. Early Axial Spondyloarthritis Detection: Two-Year Follow-up of the Sp-EYE Study on Acute Anterior Uveitis and Chronic Back Pain Screening. J Clin Rheumatol. 2025 Aug 1;31(5):e49-e57. doi: 10.1097/RHU.0000000000002202. Epub 2025 Feb 14. PMID: 39947898. | Not a referral strategy |
| Jamal M, Korver AM, Kuijper M, Lopes Barreto D, Appels CWY, Spoorenberg APL, Koes BW, Hazes JMW, Hoeven LV, Weel AEAM. The IMPACT study: A clustered randomized controlled trial to assess the effect of a referral algorithm for axial spondyloarthritis. PLoS One. 2020 Jan 28;15(1):e0227025. doi: 10.1371/journal.pone.0227025. PMID: 31990912; PMCID: PMC6986702. | Wrong study design |
| Kiltz U, Rudwaleit M, Sieper J, Braun J; German Society for Rheumatology. DGRh-S3-Leitlinie Axiale Spondyloarthritis inklusive Morbus Bechterew und Frühformen: 4 Klassifikations- und Diagnosekriterien [German Society for Rheumatology S3 guidelines on axial spondyloarthritis including Bechterew's disease and early forms: 4 Classification and diagnostic criteria]. Z Rheumatol. 2014 Sep;73 Suppl 2:40-3. German. doi: 10.1007/s00393-014-1429-5. PMID: 25181972. | Wrong study design |
| Laires, P. A.; Dezerto, R.; Mesquita, R.; Fonseca, J. E.; Cernadas, R.; Miranda, L. C.; Silva, J. C.; Costa, L.; Nogueira, A. M. Effectiveness of a referral program for early arthritis diagnosis at primary care centers in portugal-preliminary results from the siara study. Value in health 2015;18(7):A658 | Conference abstract |
| Lau IS, Gun SC, Yeap SS, Mohd Zain M, Mohd Yusoof H, Sargunan S, Yahya F. Algorithm for the referral of patients with inflammatory back pain from primary care in Malaysia. Malays Fam Physician. 2021 May 27;16(2):2-6. doi: 10.51866/rv1206. PMID: 34386157; PMCID: PMC8346753. | Wrong study design |
| Lebughe Litite P, Westhovens R, Nkodila A, Malemba JJ, de Vlam K. Development and validation of a screening tool for SPondyloArthritis Screening in Sub-Saharan Africa: SpASSS questionnaire. BMC Med Res Methodol. 2023 Jun 21;23(1):145. doi: 10.1186/s12874-023-01966-w. PMID: 37344764; PMCID: PMC10286346. | Wrong patient population |
| Liao, Z.T.; Gu, J.N.; Wu, J.; Wei, Q.J. Screening and verification of questionnaire indexes for epidemiological studies on spondyloarthropathy. Zhonghua liu xing bing xue za zhi = Zhonghua liuxingbingxue zazhi 2007;28(5):495-498 | Wrong patient population |
| Muñoz-Fernández S, de Miguel E, Cobo-Ibáñez T, Carmona L, Steiner M, Descalzo MA, Ferreira A, Balsa A, Martín-Mola E; ESPIDEP Study Group. Early spondyloarthritis: results from the pilot registry ESPIDEP. Clin Exp Rheumatol. 2010 Jul-Aug;28(4):498-503. Epub 2010 Aug 30. PMID: 20659413. | Wrong study design |
| Myasoutova, L.I.; Lapshina, S.A.; Erdes, S.F.; Salihov, I.G. Efficiency of training programs aimed at ankylosing spondylitis early detection for primary care doctors in Kazan. International Journal of Pharmacy and Technology 2016;8(2):13737-13745 | Conference abstract |
| Poddubnyy D, Callhoff J, Spiller I, Listing J, Braun J, Sieper J, Rudwaleit M. Diagnostic accuracy of inflammatory back pain for axial spondyloarthritis in rheumatological care. RMD Open. 2018 Dec 5;4(2):e000825. doi: 10.1136/rmdopen-2018-000825. PMID: 30713715; PMCID: PMC6336095. | Strategy not for screening |
| Poddubnyy D, van Tubergen A, Landewé R, Sieper J, van der Heijde D; Assessment of SpondyloArthritis international Society (ASAS). Development of an ASAS-endorsed recommendation for the early referral of patients with a suspicion of axial spondyloarthritis. Ann Rheum Dis. 2015 Aug;74(8):1483-7. doi: 10.1136/annrheumdis-2014-207151. Epub 2015 May 19. PMID: 25990288. | Wrong study design |
| Prati C, Verhoeven F, Wendling D. Reducing diagnostic delay in axial spondyloarthritis. Joint Bone Spine. 2025 Jul;92(4):105852. doi: 10.1016/j.jbspin.2025.105852. Epub 2025 Feb 1. PMID: 39900198. | Wrong study design |
| Zeidler, H.; Mau, R.; Freyschmidt, J. Evaluation of early diagnostic criteria including HLA-B27 for ankylosing spondylitis in a follow-up study. Zeitschrift fur Rheumatologie 1985;44(6):249-253 | Strategy not for screening |
