# Supplementary material for: Performance of referral strategies for patients with suspected axial spondyloarthritis: a systematic review with meta-analysis
Source: eClinicalMedicine. 2026 Aug 26;99:104167. doi: 10.1016/j.eclinm.2026.104167 (PMC13543815; doi:10.1016/j.eclinm.2026.104167)
Supplement: Supplementary file 6 [file mmc4.docx]

**SUPPLEMENTARY MATERIAL 4 – STUDY CHARACTERISTICS**

| **First author (year)** | **Study design** | **Country** | **Setting** | **Inclusion criteria** | **Exclusion criteria** | **Recruitment period** |
| --- | --- | --- | --- | --- | --- | --- |
| Abawi (2017) | Cohort study | Europe countries | Secondary care rheumatology setting (patients referred from primary care and other specialties) | Patients aged ≥ 16 years with chronic back pain almost daily, with a duration duration ≥ 3 months but ≤ 2 years, and an age of onset < 45 years | NI | NI |
| Baraliakos (2020) | Cohort study | Germany | Primary care–based referral setting (pre-selected patients with chronic back pain referred by general practitioner and orthopaedic specialists to rheumatology centres) | Patients aged ≤ 45 years with chronic LBP | NI | NI |
| Bentin (2012) | Cohort study | Belgium | Primary care–based referral setting (pre-selected patients with chronic back pain referred by general practitioners and physical medicine specialists to rheumatology centres) | Patients aged ≤ 45 years with > 3 months of LBP | NI | From June 2008 to January 2010 |
| Brandt (2007) | Cohort study | Germany | Primary care–based referral setting (pre-selected patients with chronic back pain referred by orthopaedists and primary-care physicians to a specialist rheumatology clinic) | Patients aged ≤ 45 years with chronic (> 3 months) LBP | NI | From July 2004 to December 2005 |
| Braun (2011) | Clinical trial | Germany | Primary care–based stratified referral setting (patients with chronic back pain recruited by orthopaedists and selectively referred to rheumatologists) | Patients with chronic back pain almost daily, with a duration > 2 months but < 10 years, and an age of onset between 16 and 45 years | NI | From April 2007 to June 2009 |
| Braun (2013) | Case ascertainment trial | Germany | Primary care–based stratified referral setting (patients with chronic back pain recruited by orthopaedists and selectively referred to rheumatologists) | Patients with chronic back pain almost daily, with a duration > 2 months but < 10 years, and an age of onset between 16 and 45 years | NI | From April 2007 to June 2009 |
| Braun (2019) | Cohort study | Germany | Primary care–based referral setting (pre-selected patients with chronic back pain identified by orthopaedists and referred to rheumatologists) | Patients aged > 18 years with ≥ 3 months back pain and age an age of symptoms onset < 45 years | NI | NI |
| Danve (2025) | Cohort study | United States | Community-based self-referral setting (patients with chronic back pain recruited via electronic patient portal and social media and referred to a rheumatology clinic) | Patients aged < 45 years with ≥ 3 months of gradual-onset back pain | Confirmed diagnosis of SpA or obvious alternative aetiology for back pain | From April 2019 to February 2022 |
| Hamilton (2013) | Case – control study | England | Secondary care rheumatology setting (pre-diagnosed patients recruited from hospital rheumatology clinics for screening tool validation) | Patients aged 18 to 60 years with chronic back pain | Patients unable to communicate in English, taking TNF inhibitors, or having a confounding spinal diagnosis | NI |
| Hermann (2009) | Cohort study | Austria | Primary care–based referral setting (patients pre-selected by general practitioners and referred to an early spondyloarthritis clinic) | Patients aged < 45 years | NI | NI |
| Juanola (2013) | Cohort study | Spain | Primary care–based referral setting (patients with chronic back pain referred from primary care centres to rheumatology departments according to predefined referral strategies) | Patients aged ≤ 45 years with chronic (> 3 months) back pain | Prior diagnosis of axSpA or r-axSpA | NI |
| Maksymowych (2025) (SASPIC-1) | Cohort study | Canada | Specialist non-rheumatology referral setting (patients with chronic back pain recruited from dermatology, ophthalmology, and gastroenterology clinics and referred to rheumatologists) | Patients aged ≤ 45 years with chronic (> 3 months) back pain and/or buttock pain, diagnosed with psoriasis, acute anterior uveitis, or inflammatory bowel disease. | Diagnosis axSpA, history of recent spinal trauma, contraindication to or inability to have MRI evaluation, and treatment with any biologic or targeted synthetic disease-modifying antirheumatic drug within 12 weeks before screening | From February 2013 to March 2018 |
| Maksymowych (2025) (SASPIC-2) | Cohort study | Canada | Specialist non-rheumatology referral setting (patients with chronic back pain recruited from dermatology, ophthalmology, and gastroenterology clinics and referred to rheumatologists) | Patients aged ≤ 45 years with chronic (> 3 months) back pain and/or buttock pain, diagnosed with psoriasis, acute anterior uveitis, or inflammatory bowel disease. | Diagnosis axSpA, history of recent spinal trauma, contraindication to or inability to have MRI evaluation, and treatment with any biologic or targeted synthetic disease-modifying antirheumatic  drug within 12 weeks before screening | From May 2018 to September 2022 |
| Passalent (2022) | Cohort study | Canada | Interprofessional primary care–based triage setting (patients with chronic back pain referred from primary care to an advanced practice spine assessment program before rheumatology evaluation) | Patients aged ≥ 18 years with back-dominant pain | NI | NI |
| Poddubnyy (2011) | Cohort study | Germany | Primary care–based referral setting (patients with chronic back pain pre-selected by general practitioners and orthopaedists using predefined referral strategies and referred to rheumatologists) | Patients aged ≤ 45 years with chronic (> 3 months) back pain | No confirmed diagnosis of axSpA or r-axSpA | From June 2007 to August 2009 |
| Proft (2020) | Cohort study | Germany | Mixed referral setting (patients with chronic back pain self-referred via an online tool or referred by physicians to a specialized rheumatology center) | Patients aged < 45 years with > 3 months of back pain | NI | From September 2016 to September 2017 |
| Sepriano (2019) | Cross – sectional study | Portugal | Population-based screening setting (randomly selected participants from the general population assessed through a nationwide epidemiologic study) | Patients aged ≥ 18 years with positive screening for ≥ 1 rheumatic and musculoskeletal disease | NI | From September 2011 to December 2013 |
| Sieper (2012) | Cohort study | 16 countries (12 in Europe, plus Canada, India, Israel and Venezuela) | Primary care–based referral setting (patients with chronic back pain identified at primary care referral sites and referred to rheumatologists according to predefined referral strategies) | Patients aged < 45 years with chronic (> 3 months) back pain | Established diagnosis of SpA or a clinically significant condition that interfered with the study evaluations according to the referring physician | From September 2008 to May 2010 |
| Tant (2017) | Cross – sectional study | Belgium | Non-rheumatology specialist referral setting (patients with chronic back pain screened by physical medicine and rehabilitation physicians, orthopaedic specialists, and ophthalmologists and referred to rheumatologists) | Patients aged 18 to 45 years with chronic (> 3 months) back pain at night | Diagnosis of SpA or r-axSpA | From February 2011 to June 2013 |
| Van Hoeven (2014) | Cross–sectional study | Netherlands | Primary care recruitment setting (patients with chronic low back pain identified directly from general practitioner databases) | Patients aged 20 to 45 years with LBP for more than 12 weeks | Trauma and contraindications for MRI | From January 2010 to June 2010 |
| Van Hoeven (2015) | Cross–sectional study | Netherlands | Primary care recruitment setting (patients with chronic low back pain identified directly from general practitioner databases) | Patients aged 18 to 45 years with LBP (without radiation) for more than 12 weeks | Contraindications for MRI, explainable cause for the back pain (such as trauma) | From June 2011 to June 2012 |
| Weisman (2010) | Case – control study | United States | Case–control development setting (patients with axSpA from established databases and chronic back pain controls recruited from clinics, workplaces, and an online platform) | Case: axSpA patients  Controls: chronic back pain | NI | NI |
| Xiang (2022) | Cross–sectional study | Singapore | Secondary care rheumatology referral setting (consecutive patients newly referred to a tertiary rheumatology clinic for evaluation of autoimmune rheumatic diseases) | Patients aged ≥ 21 years referred for an autoimmune rheumatic disease | No prior diagnosis of autoimmune rheumatic diseases | From April 2018 to November 2019 |
| Ziade (2023) | Cohort study | Middle Eastern Arab countries | Multispecialty first-line referral setting (patients with suspicious chronic low back pain referred by primary care physicians, medical specialists, or self-referral to rheumatology evaluation) | Patients aged 18 to 45 years presenting with chronic (≥ 3 months) LBP of suspicious nature. Suspicious LBP was defined as a chronic LBP that, in the opinion and routine practice of the referring physician, needed a referral to rheumatology | Other inflammatory disease, a known diagnosis of axSpA, prior exposure to biological therapies, current corticosteroid therapy, contraindication to MRI, and pregnancy | From April 2020 to April 2022 |
