# Supplementary material for: Performance of referral strategies for patients with suspected axial spondyloarthritis: a systematic review with meta-analysis
Source: eClinicalMedicine. 2026 Aug 26;99:104167. doi: 10.1016/j.eclinm.2026.104167 (PMC13543815; doi:10.1016/j.eclinm.2026.104167)
Supplement: Supplementary file 7 [file mmc5.docx]

**SUPPLEMENTARY MATERIAL 5 – DEVELOPMENT STUDIES CHARACTERISTICS**

| **Referral strategy** | **First author (year)** | **Modeling strategy** | **Sample size estimation** | **Method for selection of predictors for inclusion in multivariable modeling** | **Method for selection of predictors during multivariable modeling** | **Method for internal validation** |
| --- | --- | --- | --- | --- | --- | --- |
| ASAS referral criteria by Sepriano | Sepriano (2019) | Clinically selected predictors (non-statistical) | No | NI | NA | Not performed |
| ASAS referral criteria by Ziade | Ziade (2023) | Clinically selected predictors (non-statistical) | No | NI | NA | Not performed |
| A-tool | Danve (2025) | Clinically selected predictors based on a literature review | No | NI | NA | Not performed |
| AWARE ≥ 1/5 | Braun (2019) | Clinically selected predictors (non-statistical) | No | NI | NA | Not performed |
| AWARE ≥ 2/5 – Braun IBP | Braun (2011) | Clinically selected predictors (non-statistical) | No | NI | NA | Not performed |
| AWARE ≥ 3/5 | Braun (2019) | Clinically selected predictors (non-statistical) | No | NI | NA | Not performed |
| Baraliakos combination I | Baraliakos (2020) | Best combinations of features compared | No | NI | NA | Not performed |
| Baraliakos combination II | Baraliakos (2020) | Best combinations of features compared | No | NI | NA | Not performed |
| Baraliakos combination III | Baraliakos (2020) | Best combinations of features compared | No | NI | NA | Not performed |
| Brandt I plus imaging | Brandt (2007) | Clinically selected predictors (non-statistical) | No | NA | NA | Not performed |
| Braun 2-step | Braun (2013) | Best combinations of features compared | No | NI | NA | Not performed |
| Braun 2-step modified | Sepriano (2019) | Clinically selected predictors (non-statistical) | No | NI | NA | Not performed |
| CaFaSpA | Van Hoeven (2014) | Logistic regression | No | Expert opinion and univariate association | Backwards stepwise selection | Bootstrapping (200 bootstrap samples) |
| CaFaSpA ≥ 1.5 | Sepriano (2019) | Clinically selected predictors (non-statistical) | No | NI | NA | Not performed |
| Calin’s criteria for IBP | Hermann (2009) | Clinically selected predictors (non-statistical) | No | NA | NA | Not performed |
| EpiReumaPt | Sepriano (2019) | Clinically selected predictors (non-statistical) | No | NI | NA | Not performed |
| Hamilton screening tool | Hamilton (2023) | Logistic regression | Yes, but inappropriate | Variables selected from the Calin, Berlin, and ASAS IBP criteria | NI | Not performed |
| Hamilton screening tool (Xiang version) | Xiang (2022) | Clinically selected predictors (non-statistical) | No | NA | NA | Not performed |
| Hermann model | Hermann (2009) | Logistic regression | No | NI | Stepwise selection | Not performed |
| MASTER strategy 1 | Poddubnyy (2011) | Clinically selected predictors (non-statistical) | No | NA | NA | Not performed |
| MASTER strategy 2 | Poddubnyy (2011) | Clinically selected predictors (non-statistical) | No | NA | NA | Not performed |
| MASTER modified | Sepriano (2019) | Clinically selected predictors (non-statistical) | No | NI | NA | Not performed |
| OptiRef | Proft (2020) | Clinically selected predictors (non-statistical) | No | NA | NA | Not performed |
| Passalent screening tool | Passalent (2022) | Clinically selected predictors (non-statistical) | No | NA | NA | Not performed |
| RADAR strategy 1 | Sieper (2012) | Clinically selected predictors (non-statistical) | No | No | No | Not performed |
| RADAR strategy 2 | Sieper (2012) | Clinically selected predictors (non-statistical) | No | No | No | Not performed |
| RADAR by Bentin 1/3 | Bentin (2012) | Clinically selected predictors (non-statistical) | No | NA | NA | Not performed |
| RADAR by Bentin 2/6 | Bentin (2012) | Clinically selected predictors (non-statistical) | No | NA | NA | Not performed |
| SUSPECT | Tant (2017) | Clinically selected predictors (non-statistical) | No | NI | NA | Not performed |
| Weisman model | Weisman (2010) | Logistic regression | Yes, but inappropriate | Literature review, expert opinion, univariate association | Best subset selection | Split – sample approach (70% development, 30% validation) |
