# Supplementary material for: Performance of referral strategies for patients with suspected axial spondyloarthritis: a systematic review with meta-analysis
Source: eClinicalMedicine. 2026 Aug 26;99:104167. doi: 10.1016/j.eclinm.2026.104167 (PMC13543815; doi:10.1016/j.eclinm.2026.104167)
Supplement: Supplementary file 8 [file mmc6.docx]

**SUPPLEMENTARY MATERIAL 6 – PERFORMANCE MEASURES**

| **Referral strategy** | **First author (year) and type of performance** | **Reference standard** | **n** | **% axSpA** | **Discrimination**  **measures** | **Calibration**  **measures** | **Classification**  **measures** | | | | | |
| --- | --- | --- | --- | --- | --- | --- | --- | --- | --- | --- | --- | --- |
|  |  |  |  |  |  |  | SE | SP | PPV | NPV | LR + | LR - |
| ASAS referral criteria | Abawi (2017)  External validation | Rheumatologist’s diagnosis | 261 | 40.9% |  |  | 98% | 18% |  |  | 1.2 | 0.11* |
|  | Abawi (2017)  External validation | ASAS classification criteria | 261 | 40.9% |  |  | 100% | 16% |  |  | 1.2 | 0.01* |
|  | Maksymowych (2025) (SASPIC-1)  External validation | Rheumatologists’ diagnosis | 212 | 51.9% |  |  | 66.4% | 79.4% |  |  | 3.22* | 0.42* |
|  | Maksymowych (2025) (SASPIC-2)  External validation | Rheumatologists’ diagnosis | 151 | 36.4% |  |  | 85.5% | 82.3% |  |  | 4.83* | 0.18* |
| ASAS referral criteria by Sepriano | Sepriano (2019)  Apparent performance | Rheumatologists’ diagnosis (consensus across three clinicians) | 3877 | 2.4% | AUC 57.4% |  | 21.4% | 93.4% | 5.0 |  | 3.2 | 0.8 |
| ASAS referral criteria by Ziade | Ziade (2023)  Apparent performance | Rheumatologist’s diagnosis | 471 | 52.9% |  |  | 100% | 15.8% | 57.1% | 100% | 1.2 | 0.01* |
| A-tool | Danve (2025)  Apparent performance | Rheumatologist’s diagnosis | 86 | 33.7% | AUC 66% |  |  |  |  |  |  |  |
| AWARE ≥ 1/5 | Braun (2019)  Apparent performance | Rheumatologist’s diagnosis | 343 | NI |  |  | 100% (95% CI: 97.3 to 100) | 0.97% (95% CI: 0.12 to 3.46) |  |  | 1.01* | 0.01* |
| AWARE ≥ 2/5 - Braun IBP | Abawi (2017)  External validation | Rheumatologist’s diagnosis | 261 | 40.9% |  |  | 79% | 39% |  |  | 1.29 | 0.54* |
|  | Abawi (2017)  External validation | ASAS classification criteria | 261 | 40.9% |  |  | 82% | 38% |  |  | 1.33 | 0.47* |
|  | Braun (2011)  Apparent performance | Rheumatologist’s diagnosis | 322 | 35.1% | AUC 71.3% |  | 96.5% | 17.0% |  |  | 1.16* | 0.21* |
|  | Braun (2019)  External validation | Rheumatologist’s diagnosis | 343 | 41.1% |  |  | 99.3% (95% CI: 96 to 100) | 1.94% (95% CI: 0.53 to 4.90) |  |  | 1.01* | 0.36* |
|  | Sepriano (2019)  External validation | Rheumatologists’ diagnosis (consensus across three clinicians) | 3877 | 2.4% | AUC 63.1% |  | 47.5% | 78.7% | 3.5% |  | 2.2 | 0.7 |
|  | Ziade (2023)  External validation | Rheumatologist’s diagnosis | 471 | 52.9% |  |  | 98.4% | 15.8% | 56.6% | 89.7% | 1.2 | 0.1* |
| AWARE ≥ 3/5 | Braun (2019)  Apparent performance | Rheumatologist’s diagnosis | 343 | NI |  |  | 93.4% (95% CI: 87.9 to 97) | 14.6% (95% CI: 10.1 to 20.1) |  |  | 1.09* | 0.45* |
| Baraliakos combination I | Baraliakos (2020)  Apparent performance | Rheumatologist’s diagnosis | 326 | 14.1% |  |  | 91% | 67% | 31% | 98% | 2.78 | 0.13 |
| Baraliakos combination II | Baraliakos (2020)  Apparent performance | Rheumatologist’s diagnosis | 326 | 14.1% |  |  | 78% | 85% | 46% | 96% | 5.10 | 0.26 |
| Baraliakos combination III | Baraliakos (2020)  Apparent performance | Rheumatologist’s diagnosis | 326 | 14.1% |  |  | 74% | 85% | 45% | 95% | 5.05 | 0.31 |
| Brandt I | Abawi (2017)  External validation | Rheumatologist’s diagnosis | 261 | 40.9% |  |  | 98% | 11% |  |  | 1.1 | 0.18 |
|  | Abawi (2017)  External validation | ASAS classification criteria | 261 | 40.9% |  |  | 100% | 10% |  |  | 1.12 | 0.01* |
|  | Sepriano (2019)  External validation | Rheumatologists’ diagnosis (consensus across three clinicians) | 3877 | 2.4% | AUC 64.3% |  | 49.2% | 79.3% | 3.7% |  | 2.4 | 0.6 |
|  | Ziade (2023)  External validation | Rheumatologist’s diagnosis | 471 | 52.9% |  |  | 99.2% | 15.8% | 56.9% | 94.6% | 1.2 | 0.05* |
| Brandt I plus imaging | Brandt (2007)  Apparent performance | Rheumatologist’s diagnosis | 350 | 45.4% |  |  |  |  | 45.4% |  |  |  |
|  | Proft (2020)  External validation | Rheumatologist’s diagnosis | 181 | 39.2% |  |  |  |  | 39.2% |  |  |  |
| Brandt II | Abawi (2017)  External validation | Rheumatologist’s diagnosis | 261 | 40.9% |  |  | 93% | 25% |  |  | 1.23 | 0.28* |
|  | Abawi (2017)  External validation | ASAS classification criteria | 261 | 40.9% |  |  | 97% | 24% |  |  | 1.29 | 0.13* |
|  | Sepriano (2019)  External validation | Rheumatologists’ diagnosis (consensus across three clinicians) | 3877 | 2.4% | AUC 60.1% |  | 27.7% | 92.4% | 5.6% |  | 3.7 | 0.8 |
|  | Ziade (2023)  External validation | Rheumatologist’s diagnosis | 471 | 52.9% |  |  | 95.2% | 51.8% | 68.9% | 90.6% | 2.0 | 0.09* |
| Brandt III | Abawi (2017)  External validation | Rheumatologist’s diagnosis | 261 | 40.9% |  |  | 74% | 60% |  |  | 1.86 | 0.43* |
|  | Abawi (2017)  External validation | ASAS classification criteria | 261 | 40.9% |  |  | 90% | 62% |  |  | 2.37 | 0.16* |
|  | Sepriano (2019)  External validation | Rheumatologists’ diagnosis (consensus across three clinicians) | 3877 | 2.4% | AUC 53.2% |  | 7.9% | 98.4% | 7.6% |  | 5.1 | 0.9 |
|  | Ziade (2023)  External validation | Rheumatologist’s diagnosis | 471 | 52.9% |  |  | 73.1% | 76.1% | 77.4% | 71.6% | 3.1 | 0.35* |
| Braun 2-step | Abawi (2017)  External validation | Rheumatologist’s diagnosis | 261 | 40.9% |  |  | 75% | 59% |  |  | 1.83 | 0.42* |
|  | Abawi (2017)  External validation | ASAS classification criteria | 261 | 40.9% |  |  | 90% | 60% |  |  | 2.27 | 0.17* |
|  | Braun (2013)  Apparent performance | Rheumatologist’s diagnosis | 298 | 36.9% |  |  | 80.4% | 75.4% | 64.6% | 87.2% | 3.27 | 0.26 |
| Braun 2-step alt. | Abawi (2017)  External validation | Rheumatologist’s diagnosis | 261 | 40.9% |  |  | 65% | 69% |  |  | 2.1 | 0.51* |
|  | Abawi (2017)  External validation | ASAS classification criteria | 261 | 40.9% |  |  | 86% | 73% |  |  | 3.13 | 0.19* |
|  | Baraliakos (2020)  External validation | Rheumatologist’s diagnosis | 326 | 14.1% |  |  | 87% | 56% | 24.8% | 96.4% | 1.98* | 0.23* |
| Braun 2-step modified | Sepriano (2019)  Apparent performance | Rheumatologists’ diagnosis (consensus across three clinicians) | 3877 | 2.4% | AUC 55.4% |  | 15.1% | 95.7% | 5.3% |  | 3.5 | 0.9 |
| CaFaSpA | Van Hoeven (2014)  Internal validation | ASAS classification criteria | 364 | 24.8% |  |  | 83% | 59% |  |  | 2.02* | 0.29* |
|  | Van Hoeven (2015)  External validation | ASAS classification criteria | 579 | 16.4% | C-statistic 0.70 (95% CI: 0.64 to 0.75) | Slope 0.77 (95% CI: 0.49 to 1.06)  Intercept -0.48 (95% CI: - 0.73 to - 0.25) |  |  | 30.2% |  |  |  |
| CaFaSpA ≥ 1 | Abawi (2017)  External validation | Rheumatologist’s diagnosis | 261 | 40.9% |  |  | 87% | 31% |  |  | 1.25 | 0.42* |
|  | Abawi (2017)  External validation | ASAS classification criteria | 261 | 40.9% |  |  | 94% | 31% |  |  | 1.35 | 0.19* |
|  | Sepriano (2019)  External validation | Rheumatologists’ diagnosis (consensus across three clinicians) | 3877 | 2.4% | AUC 63% |  | 56.3% | 69.7% | 2.9% |  | 1.9 | 0.6 |
|  | Ziade (2023)  External validation | Rheumatologist’s diagnosis | 471 | 52.9% |  |  | 81.1% | 58.1% | 68.5% | 73.3% | 1.9 | 0.33* |
| CaFaSpA ≥ 1.5 | Sepriano (2019)  Apparent performance | Rheumatologists’ diagnosis (consensus across three clinicians) | 3877 | 2.4% | AUC 63% |  | 43.9% | 82.1% | 3.8% |  | 2.5 | 0.7 |
| CaFaSpA ≥ 2 | Abawi (2017)  External validation | Rheumatologist’s diagnosis | 261 | 40.9% |  |  | 51% | 74% |  |  | 1.98 | 0.66* |
|  | Abawi (2017)  External validation | ASAS classification criteria | 261 | 40.9% |  |  | 61% | 74% |  |  | 2.35 | 0.53* |
|  | Sepriano (2019)  Apparent performance | Rheumatologists’ diagnosis (consensus across three clinicians) | 3877 | 2.4% | AUC 55.2% |  | 15.2% | 95.2% | 4.9% |  | 3.1 | 0.9 |
| Calin’s criteria for IBP | Abawi (2017)  External validation | Rheumatologist’s diagnosis | 261 | 40.9% |  |  | 83% | 30% |  |  | 1.19 | 0.57 |
|  | Abawi (2017)  External validation | ASAS classification criteria | 261 | 40.9% |  |  | 85% | 29% |  |  | 1.19 | 0.52* |
|  | Hermann (2009)  Apparent performance | Rheumatologist’s diagnosis | 92 | 32.6% |  |  |  |  |  |  | 1.4 (95% CI: 1.1 to 1.6) | 0.3 (95% CI: 0.1 to 0.7) |
|  | Sepriano (2019)  External validation | Rheumatologists’ diagnosis (consensus across three clinicians) | 3877 | 2.4% | AUC 57.8% |  | 22.4% | 93.2% | 5.1% |  | 3.3 | 0.8 |
|  | Ziade (2023)  External validation | Rheumatologist’s diagnosis | 471 | 52.9% |  |  | 89.6% | 40.5% | 62.8% | 77.6% | 1.5 | 0.26* |
| EpiReumaPt | Sepriano (2019)  Apparent performance | Rheumatologists’ diagnosis (consensus across three clinicians) | 3877 | 2.4% | AUC 69.9% |  | 72.1% | 67.7% | 3.5% |  | 2.2 | 0.4 |
| Hamilton screening tool | Hamilton (2013)  Apparent performance | Modified New York criteria | 190 | 40.0% | AUC 91% (95% CI: 87% to 96%) |  | 75.6% | 87.9% |  |  | 6.25* | 0.28* |
|  | Xiang (2022)  External validation | Rheumatologist’s diagnosis | 1418 | 3.2% |  |  | 34.8% (95% CI: 21.4 to 50.2) | 93.7% (95% CI: 92.3 to 95.0) | 15.7% (95% CI: 9.2 to 24.2) | 97.7% (95% CI: 96.8 to 98.5) | 5.52* | 0.7* |
| Hamilton screening tool (Xiang version) | Xiang (2022)  Apparent performance | Rheumatologist’s diagnosis | 1418 | 3.2% |  |  | 82.6% (95% CI: 68.6 to 92.2) | 37.2% (95% CI: 34.7 to 39.9) | 4.2% (95% CI: 3.0 to 5.8) | 98.5% (95% CI: 97.0 to 99.3) | 1.32* | 0.47* |
| Hermann model | Hermann (2009)  Apparent performance | Rheumatologist’s diagnosis | 92 | 32.6% | AUC 98% |  | 90% | 95% |  |  | 16.8 (95% CI: 5.6 to 50.8) | 0.11* |
| MASTER strategy 1 | Poddubnyy (2011)  Apparent performance | Rheumatologist’s diagnosis | 318 | 41.8% |  |  |  |  | 41.8% |  |  |  |
| MASTER strategy 2 | Poddubnyy (2011)  Apparent performance | Rheumatologist’s diagnosis | 242 | 41.8% |  |  |  |  | 36.8% |  |  |  |
|  | Ziade (2023)  External validation | Rheumatologist’s diagnosis | 471 | 52.9% |  |  | 67.1% | 79.7% | 78.8% | 68.3% | 3.3 | 0.41* |
| MASTER without imaging | Abawi (2017)  External validation | Rheumatologist’s diagnosis | 261 | 40.9% |  |  | 64% | 76% |  |  | 2.68 | 0.47* |
|  | Abawi (2017)  External validation | ASAS classification criteria | 261 | 40.9% |  |  | 76% | 75% |  |  | 3.0 | 0.32* |
| MASTER modified | Sepriano (2019)  Apparent performance | Rheumatologists’ diagnosis (consensus across three clinicians) | 3877 | 2.4% | AUC 62.2% |  | 36.7% | 87.7% | 4.6% |  | 3.0 | 0.7 |
| OptiRef | Proft (2020)  Apparent performance | Rheumatologist’s diagnosis | 180 | 19.4% |  |  | 100% | 12% | 21% | 100% | 1.14 | 0.01 |
| Passalent screening tool | Passalent (2022)  Apparent performance | Rheumatologist’s diagnosis | 405 | 15.5% |  |  | 73% (95% CI: 65 to 80) | 44% (95% CI: 38 to 50) | 41% (95% CI: 35 to 47) | 75% (95% CI: 68 to 82) | 1.3* | 0.61* |
| RADAR strategy 1 | Juanola (2013)  External validation | Rheumatologist’s diagnosis | 28 | 28.6% |  |  |  |  | 28.5% |  |  |  |
|  | Sieper (2012)  Apparent performance | Rheumatologist’s diagnosis | 504 | 34.9% |  |  |  |  | 34.9% |  |  |  |
| RADAR strategy 2 | Juanola (2013)  External validation | Rheumatologist’s diagnosis | 59 | 25.4% |  |  |  |  | 25.4% |  |  |  |
|  | Sieper (2012)  Apparent performance | Rheumatologist’s diagnosis | 568 | 38.9% |  |  |  |  | 38.9% |  |  |  |
|  | Ziade (2023)  External validation | Rheumatologist’s diagnosis | 471 | 52.9% |  |  | 75.5% | 75.2% | 77.4% | 73.2% | 3.0 | 0.33* |
| RADAR without imaging | Abawi (2017)  External validation | Rheumatologist’s diagnosis | 261 | 40.9% |  |  | 79% | 63% |  |  | 2.12 | 0.33* |
|  | Abawi (2017)  External validation | ASAS classification criteria | 261 | 40.9% |  |  | 90% | 62% |  |  | 2.34 | 0.16* |
| RADAR 2/3 | Abawi (2017)  External validation | Rheumatologist’s diagnosis | 261 | 40.9% |  |  | 44% | 82% |  |  | 2.51 | 0.68* |
|  | Abawi (2017)  External validation | ASAS classification criteria | 261 | 40.9% |  |  | 39% | 76% |  |  | 1.66 | 0.8* |
|  | Ziade (2023)  External validation | Rheumatologist’s diagnosis | 471 | 52.9% |  |  | 55% | 81.1% | 76.5% | 61.6% | 2.9 | 0.55* |
| RADAR by Bentin 1/3 | Bentin (2012)  Apparent performance | Rheumatologist’s diagnosis | 73 | 26.0% |  |  |  |  | 26% |  |  |  |
| RADAR by Bentin 2/6 | Bentin (2012)  Apparent performance | Rheumatologist’s diagnosis | 65 | 36.9% |  |  |  |  | 36.9% |  |  |  |
| SUSPECT | Tant (2017)  Apparent performance | Rheumatologist’s diagnosis | 85 | 43.5% |  |  | 59.4% | 62.5% | 55% | 66.7% | 1.58* | 0.65* |
| Weisman model | Weisman (2010)  Apparent performance | NI | 316 | 32.3% | AUC 94% |  | 69.6% | 99.1% | 79.7% | 98.4% | 77.33* | 0.31* |
|  | Weisman (2010)  Internal validation | NI | 135 | 31.8% |  |  | 67.4% | 94.6% |  |  | 12.48* | 0.34* |

AUC: area under the receiver operating characteristic curve, SE, sensitivity, SP: specificity, PPV: positive predictive value, NPV: negative predictive value, LR+: positive likelihood ratio, LR−: negative likelihood ratio, CI: confidence interval, NI: not indicated.

*Likelihood ratios calculated by the review authors from reported sensitivity and specificity using standard formulas as reported in the methods section of the manuscript
