# Supplementary material for: Performance of referral strategies for patients with suspected axial spondyloarthritis: a systematic review with meta-analysis
Source: eClinicalMedicine. 2026 Aug 26;99:104167. doi: 10.1016/j.eclinm.2026.104167 (PMC13543815; doi:10.1016/j.eclinm.2026.104167)
Supplement: Supplementary file 9 [file mmc7.docx]

**SUPPLEMENTARY MATERIAL 7**

| **Referral strategy and type of performance** | **Number of studies (axSpA/not axSpA)** | **FACTORS THAT MAY DECREASE THE QUALITY** | | | | | | **FACTORS THAT MAY INCREASE THE QUALITY** | | **RESULTS** | **Certainty of the evidence** |
| --- | --- | --- | --- | --- | --- | --- | --- | --- | --- | --- | --- |
|  |  | **Type of performance** | **Study limitations** | **Inconsistency** | **Indirectness** | **Imprecision** | **Publication bias** | **Moderate or large effect size** | **Exposure-response gradient** |  |  |
| ASAS referral criteria  Sensitivity | Meta – analysis of 3 externally validated performances measures (272/352) | EV | Very serious | Yes | No | Yes | No | No | No | 88.9% (63.3% to 97.4%) | **Very low** |
| ASAS referral criteria  Specificity | Meta – analysis of 3 externally validated performances measures (272/352) | EV | Very serious | Yes | No | Yes | No | No | No | 61.3% (24.3% to 88.7%) | **Very low** |
| ASAS referral criteria  LR+ | Meta – analysis of 3 externally validated performances measures (272/352) | EV | Very serious | Yes | No | Yes | No | No | No | 2.30 (0.85 to 6.21) | **Very low** |
| ASAS referral criteria  LR- | Meta – analysis of 3 externally validated performances measures (272/352) | EV | Very serious | Yes | No | Yes | No | No | No | 0.18 (0.04 to 0.81) | **Very low** |
| ASAS referral criteria by Sepriano  Sensitivity | 1 apparent performance measure (92/3877) | AP | Very serious | NA | Yes | Yes | Yes | No | No | 21.4% | **Very low** |
| ASAS referral criteria by Sepriano  Specificity | 1 apparent performance measure (92/3877) | AP | Very serious | NA | Yes | Yes | Yes | Yes | No | 93.4% | **Very low** |
| ASAS referral criteria by Sepriano  LR+ | 1 apparent performance measure (92/3785) | AP | Very serious | NA | Yes | Yes | Yes | No | No | 3.2 | **Very low** |
| ASAS referral criteria by Sepriano  LR- | 1 apparent performance measure (92/3785) | AP | Very serious | NA | Yes | Yes | Yes | No | No | 0.8 | **Very low** |
| ASAS referral criteria by Sepriano  AUC | 1 apparent performance measure (92/3785) | AP | Very serious | NA | Yes | Yes | Yes | No | No | 57.4% | **Very low** |
| ASAS referral criteria by Ziade  Sensitivity | 1 apparent performance measure (249/222) | AP | Very serious | NA | No | Yes | Yes | Yes | No | 100% | **Very low** |
| ASAS referral criteria by Ziade  Specificity | 1 apparent performance measure (249/222) | AP | Very serious | NA | No | Yes | Yes | No | No | 15.8% | **Very low** |
| ASAS referral criteria by Ziade  LR+ | 1 apparent performance measure (249/222) | AP | Very serious | NA | No | Yes | Yes | No | No | 1.2 | **Very low** |
| ASAS referral criteria by Ziade  LR- | 1 apparent performance measure (249/222) | AP | Very serious | NA | No | Yes | Yes | Yes | No | 0.01 | **Very low** |
| A-tool  AUC | 1 apparent performance measure (29/57) | AP | Very serious | NA | Yes | Yes | Yes | No | No | 66% | **Very low** |
| AWARE ≥ 1/5  Sensitivity | 1 apparent performance measure (140/203) | AP | Very serious | NA | No | No | Yes | Yes | No | 100% (97.3% to 100%) | **Very low** |
| AWARE ≥ 1/5  Specificity | 1 apparent performance measure (140/203) | AP | Very serious | NA | No | No | Yes | No | No | 0.97% (0.12% to 3.46%) | **Very low** |
| AWARE ≥ 1/5  LR+ | 1 apparent performance measure (140/203) | AP | Very serious | NA | No | Yes | Yes | No | No | 1.01 | **Very low** |
| AWARE ≥ 1/5  LR- | 1 apparent performance measure (140/203) | AP | Very serious | NA | No | Yes | Yes | No | No | 0.01 | **Very low** |
| AWARE ≥ 2/5 - Braun IBP  Sensitivity | Meta – analysis of 4 externally validated performance measures (589/4363) | EV | Very serious | Yes | No | Yes | No | No | No | 92.7% (61.9% to 99.0%) | **Very low** |
| AWARE ≥ 2/5 - Braun IBP  Specificity | Meta – analysis of 4 externally validated performance measures (589/4363) | EV, high | Very serious -2 | Yes | No | Yes | No | No | No | 23.8% (4.6% to 66.8%) | **Very low** |
| AWARE ≥ 2/5 - Braun IBP  LR+ | Meta – analysis of 4 externally validated performance measures (589/4363) | EV, high | Very serious -2 | Yes | No | Yes | No | No | No | 1.22 (0.76 to 1.94) | **Very low** |
| AWARE ≥ 2/5 - Braun IBP  LR- | Meta – analysis of 4 externally validated performance measures (589/4363) | EV, high | Very serious -2 | Yes | No | Yes | No | No | No | 0.30 (0.03 to 3.30) | **Very low** |
| AWARE ≥ 2/5 - Braun IBP  AUC | 1 externally validated performance measure (92/3785) | EV | Very serious | NA | Yes | Yes | Yes | No | No | 63.1% | **Very low** |
| AWARE ≥ 3/5  Sensitivity | 1 apparent performance measure (total: 343) | AP | Very serious | NA | No | No | Yes | Yes | No | 93.4% (87.9% to 97.0%) | **Very low** |
| AWARE ≥ 3/5  Specificity | 1 apparent performance measure (total: 343) | AP | Very serious | NA | No | No | Yes | No | No | 14.6% (10.1% to 20.1%) | **Very low** |
| AWARE ≥ 3/5  LR+ | 1 apparent performance measure (total: 343) | AP | Very serious | NA | No | No | Yes | Yes | No | 1.09 | **Very low** |
| AWARE ≥ 3/5  LR- | 1 apparent performance measure (total: 343) | AP | Very serious | NA | No | No | Yes | Yes | No | 0.45 | **Very low** |
| Baraliakos combination I  Sensitivity | 1 apparent performance measure (46/280) | AP | Very serious | NA | No | No | Yes | Yes | No | 91% | **Very low** |
| Baraliakos combination I  Specificity | 1 apparent performance measure (46/280) | AP | Very serious | NA | No | No | Yes | No | No | 67% | **Very low** |
| Baraliakos combination I  LR+ | 1 apparent performance measure (46/280) | AP | Very serious | NA | No | No | Yes | No | No | 2.78 | **Very low** |
| Baraliakos combination I  LR- | 1 apparent performance measure (46/280) | AP | Very serious | NA | No | No | Yes | No | No | 0.13 | **Very low** |
| Baraliakos combination II  Sensitivity | 1 apparent performance measure (46/280) | AP | Very serious | NA | No | No | Yes | No | No | 78% | **Very low** |
| Baraliakos combination II  Specificity | 1 apparent performance measure (46/280) | AP | Very serious | NA | No | No | Yes | Yes | No | 85% | **Very low** |
| Baraliakos combination II  LR+ | 1 apparent performance measure (46/280) | AP | Very serious | NA | No | No | Yes | No | No | 5.10 | **Very low** |
| Baraliakos combination II  LR- | 1 apparent performance measure (46/280) | AP | Very serious | NA | No | No | Yes | No | No | 0.26 | **Very low** |
| Baraliakos combination III  Sensitivity | 1 apparent performance measure (46/280) | AP | Very serious | NA | No | No | Yes | No | No | 74% | **Very low** |
| Baraliakos combination III  Specificity | 1 apparent performance measure (46/280) | AP | Very serious | NA | No | No | Yes | Yes | No | 85% | **Very low** |
| Baraliakos combination III  LR+ | 1 apparent performance measure (46/280) | AP | Very serious | NA | No | No | Yes | No | No | 5.05 | **Very low** |
| Baraliakos combination III  LR- | 1 apparent performance measure (46/280) | AP | Very serious | NA | No | No | Yes | No | No | 0.31 | **Very low** |
| Brandt I  Sensitivity | Meta – analysis of 3 externally validated performance measures (448/4161) | EV | Very serious | Yes | Yes | Yes | No | No | No | 95.0% (59.8% to 99.6%) | **Very low** |
| Brandt I  Specificity | Meta – analysis of 3 externally validated performance measures (448/4161) | EV | Very serious | Yes | Yes | Yes | No | No | No | 30.9% (7.2% to 72.1%) | **Very low** |
| Brandt I  LR+ | Meta – analysis of 3 externally validated performance measures (448/4161) | EV | Very serious | Yes | Yes | Yes | No | No | No | 1.38 (0.79 to 2.40) | **Very low** |
| Brandt I  LR- | Meta – analysis of 3 externally validated performance measures (448/4161) | EV | Very serious | Yes | Yes | Yes | No | No | No | 0.16 (0.01 to 2.41) | **Very low** |
| Brandt I plus imaging  PPV | 1 externally validated performance measure (71/110) | EV | Very serious | NA | Yes | Yes | Yes | No | No | 39.2% | **Very low** |
| Brandt II  Sensitivity | Meta – analysis of 3 externally validated performance measures (448/4161) | EV | Very serious | Yes | Yes | Yes | No | No | No | 82.0% (37.1% to 97.2%) | **Very low** |
| Brandt II  Specificity | Meta – analysis of 3 externally validated performance measures (448/4161) | EV | Very serious | Yes | Yes | Yes | No | No | No | 62.0% (22.8% to 90.0%) | **Very low** |
| Brandt II  LR+ | Meta – analysis of 3 externally validated performance measures (448/4161) | EV | Very serious | Yes | Yes | Yes | No | No | No | 2.16 (0.70 to 6.62) | **Very low** |
| Brandt II  LR- | Meta – analysis of 3 externally validated performance measures (448/4161) | EV | Very serious | Yes | Yes | Yes | No | No | No | 0.29 (0.05 to 1.75) | **Very low** |
| Brandt II  AUC | 1 externally validated performance measure (92/3785) | EV | Very serious | NA | Yes | Yes | Yes | No | No | 64.3% | **Very low** |
| Brandt III  Sensitivity | Meta – analysis of 3 externally validated performance measures (448/4161) | EV | Very serious | Yes | Yes | Yes | No | No | No | 46.6% (11.8% to 85.2%) | **Very low** |
| Brandt III  Specificity | Meta – analysis of 3 externally validated performance measures (448/4161) | EV | Very serious | Yes | Yes | Yes | No | No | No | 87.1% (52.5% to 97.6%) | **Very low** |
| Brandt III  LR+ | Meta – analysis of 3 externally validated performance measures (448/4161) | EV | Very serious | Yes | Yes | Yes | No | No | No | 3.60 (0.56 to 23.31) | **Very low** |
| Brandt III  LR- | Meta – analysis of 3 externally validated performance measures (448/4161) | EV | Very serious | Yes | Yes | Yes | No | No | No | 0.61 (0.25 to 1.52) | **Very low** |
| Brandt III  AUC | 1 externally validated performance measure (92/3785) | EV | Very serious | NA | Yes | No | Yes | No | No | 53.2% | **Very low** |
| Braun 2-step  Sensitivity | 1 externally validated performance measure (107/154) | EV | Very serious | NA | No | No | Yes | No | No | 75% | **Very low** |
| Braun 2-step  Specificity | 1 externally validated performance measure (107/154) | EV | Very serious | NA | No | No | Yes | No | No | 59% | **Very low** |
| Braun 2-step  LR+ | 1 externally validated performance measure (107/154) | EV | Very serious | NA | No | No | Yes | No | No | 1.83 | **Very low** |
| Braun 2-step  LR- | 1 externally validated performance measure (107/154) | EV | Very serious | NA | No | No | Yes | No | No | 0.42 | **Very low** |
| Braun 2-step alt.  Sensitivity | Meta – analysis of 2 externally validated performance measures (153/434) | EV | Very serious | Yes | No | Yes | No | No | No | 76.5% (57.4% to 88.7%) | **Very low** |
| Braun 2-step alt.  Specificity | Meta – analysis of 2 externally validated performance measures (153/434) | EV | Very serious | Yes | No | Yes | No | No | No | 62.1% (52.8% to 70.5%) | **Very low** |
| Braun 2-step alt.  LR+ | Meta – analysis of 2 externally validated performance measures (153/434) | EV | Very serious | Yes | No | Yes | No | No | No | 2.02 (1.47 to 2.76) | **Very low** |
| Braun 2-step alt.  LR- | Meta – analysis of 2 externally validated performance measures (153/434) | EV | Very serious | Yes | No | Yes | No | No | No | 0.38 (0.19 to 0.75) | **Very low** |
| Braun 2-step modified  Sensitivity | 1 externally validated performance measure (92/3785) | EV | Very serious | NA | Yes | No | Yes | No | No | 15.1% | **Very low** |
| Braun 2-step modified  Specificity | 1 externally validated performance measure (92/3785) | EV | Very serious | NA | Yes | No | Yes | Yes | No | 95.7% | **Very low** |
| Braun 2-step modified  LR+ | 1 externally validated performance measure (92/3785) | EV | Very serious | NA | Yes | No | Yes | No | No | 3.5 | **Very low** |
| Braun 2-step modified  LR- | 1 externally validated performance measure (92/3785) | EV | Very serious | NA | Yes | No | Yes | No | No | 0.9 | **Very low** |
| Braun 2-step modified  AUC | 1 externally validated performance measure (92/3785) | EV | Very serious | NA | Yes | No | Yes | No | No | 55.4% | **Very low** |
| CaFaSpA  Sensitivity | 1 internally validated performance measure (86/278) | EV | Very serious | NA | No | No | Yes | No | No | 83% | **Very low** |
| CaFaSpA  Specificity | 1 internally validated performance measure (86/278) | EV | Very serious | NA | No | No | Yes | No | No | 59% | **Very low** |
| CaFaSpA  LR+ | 1 internally validated performance measure (86/278) | EV | Very serious | NA | No | No | Yes | No | No | 2.02 | **Very low** |
| CaFaSpA  LR- | 1 internally validated performance measure (86/278) | EV | Very serious | NA | No | No | Yes | No | No | 0.29 | **Very low** |
| CaFaSpA  C-statistics | 1 externally validated performance measure (95/484) | EV, | Very serious | NA | No | No | Yes | No | No | 0.70 (0.64 to 0.75) | **Very low** |
| CaFaSpA  Calibration intercept | 1 externally validated performance measure (95/484) | EV, | Very serious | NA | No | No | Yes | No | No | -0.48 (- 0.73 to - 0.25) | **Very low** |
| CaFaSpA  Calibration slope | 1 externally validated performance measure (95/484) | EV, | Very serious | NA | No | No | Yes | No | No | 0.77 (0.49 to 1.06) | **Very low** |
| CaFaSpA ≥ 1  Sensitivity | Meta – analysis of 3 externally validated performance measures (448/4161) | EV | Very serious | Yes | No | Yes | No | No | No | 76.9% (60.5% to 87.9%) | **Very low** |
| CaFaSpA ≥ 1  Specificity | Meta – analysis of 3 externally validated performance measures (448/4161) | EV | Very serious | Yes | No | Yes | No | No | No | 53.2% (34.2% to 71.3%) | **Very low** |
| CaFaSpA ≥ 1  LR+ | Meta – analysis of 3 externally validated performance measures (448/4161) | EV | Very serious | Yes | No | Yes | No | No | No | 1.64 (1.04 to 2.59) | **Very low** |
| CaFaSpA ≥ 1  LR- | Meta – analysis of 3 externally validated performance measures (448/4161) | EV | Very serious | Yes | No | Yes | No | No | No | 0.43 (0.21 to 0.87) | **Very low** |
| CaFaSpA ≥ 1  AUC | 1 externally validated performance measure (92/3785) | EV | Very serious | NA | No | Yes | Yes | No | No | 63% | **Very low** |
| CaFaSpA ≥ 1.5  Sensitivity | 1 apparent performance measure (92/3785) | AP, | Very serious | NA | Yes | No | Yes | No | No | 43.9% | **Very low** |
| CaFaSpA ≥ 1.5  Specificity | 1 apparent performance measure (92/3785) | AP, | Very serious | NA | Yes | No | Yes | No | No | 82.1% | **Very low** |
| CaFaSpA ≥ 1.5  LR+ | 1 apparent performance measure (92/3785) | AP, | Very serious | NA | Yes | No | Yes | No | No | 2.5 | **Very low** |
| CaFaSpA ≥ 1.5  LR- | 1 apparent performance measure (92/3785) | AP, | Very serious | NA | Yes | No | Yes | No | No | 0.7 | **Very low** |
| CaFaSpA ≥ 1.5  AUC | 1 apparent performance measure (92/3785) | AP, | Very serious | NA | Yes | No | Yes | No | No | 63% | **Very low** |
| CaFaSpA ≥ 2  Sensitivity | Meta – analysis of 2 externally validated performance measures (199/3939) | EV | Very serious | Yes | No | Yes | No | No | No | 30.6% (11.3% to 60.5%) | **Very low** |
| CaFaSpA ≥ 2  Specificity | Meta – analysis of 2 externally validated performance measures (199/3939) | EV | Very serious | Yes | No | Yes | No | No | No | 88.5% (66.7% to 96.7%) | **Very low** |
| CaFaSpA ≥ 2  LR+ | Meta – analysis of 2 externally validated performance measures (199/3939) | EV | Very serious | Yes | No | Yes | No | No | No | 2.66 (0.61 to 11.56) | **Very low** |
| CaFaSpA ≥ 2  LR- | Meta – analysis of 2 externally validated performance measures (199/3939) | EV | Very serious | Yes | No | Yes | No | No | No | 0.78 (0.52 to 1.18) | **Very low** |
| CaFaSpA ≥ 2  AUC | 1 externally validated performance measure (92/3785) | EV | Very serious | NA | No | Yes | No | No | No | 55.2% | **Very low** |
| Calin’s criteria for IBP  Sensitivity | Meta – analysis of 3 externally validated performance measures (448/4161) | EV | Very serious | Yes | Yes | Yes | No | No | No | 70.1% (30.3% to 92.7%) | **Very low** |
| Calin’s criteria for IBP  Specificity | Meta – analysis of 3 externally validated performance measures (448/4161) | EV | Very serious | Yes | Yes | Yes | No | No | No | 61.4% (21.7% to 90.1%) | **Very low** |
| Calin’s criteria for IBP  LR+ | Meta – analysis of 3 externally validated performance measures (448/4161) | EV | Very serious | Yes | Yes | Yes | No | No | No | 1.82 (0.55 to 5.95) | **Very low** |
| Calin’s criteria for IBP  LR- | Meta – analysis of 3 externally validated performance measures (448/4161) | EV | Very serious | Yes | Yes | Yes | No | No | No | 0.49 (0.12 to 1.90) | **Very low** |
| Calin’s criteria for IBP  AUC | 1 externally validated performance measure (92/3785) | EV | Very serious | Yes | Yes | No | No | No | No | 55.2% | **Very low** |
| EpiReumaPt  Sensitivity | 1 apparent performance measure (92/3785) | AP | Very serious | NA | Yes | Yes | Yes | No | No | 72.1% | **Very low** |
| EpiReumaPt  Specificity | 1 apparent performance measure (92/3785) | AP | Very serious | NA | Yes | Yes | Yes | No | No | 67.7% | **Very low** |
| EpiReumaPt  LR+ | 1 apparent performance measure (92/3785) | AP | Very serious | NA | Yes | Yes | Yes | No | No | 2.2 | **Very low** |
| EpiReumaPt  LR- | 1 apparent performance measure (92/3785) | AP | Very serious | NA | Yes | Yes | Yes | No | No | 0.4 | **Very low** |
| EpiReumaPt  AUC | 1 apparent performance measure (92/3785) | AP | Very serious | NA | Yes | Yes | Yes | No | No | 69.9% | **Very low** |
| Hamilton screening tool  Sensitivity | 1 externally validated performance measure (46/1372) | EV | Very serious | NA | No | Yes | Yes, | No | No | 34.8% (21.4% to 50.2%) | **Very low** |
| Hamilton screening tool  Specificity | 1 externally validated performance measure (46/1372) | EV | Very serious | NA | No | Yes | Yes | Yes | No | 93.7% (92.3% to 95.0%) | **Very low** |
| Hamilton screening tool  LR+ | 1 externally validated performance measure (46/1372) | EV | Very serious | NA | No | Yes | Yes, | No | No | 5.52 | **Very low** |
| Hamilton screening tool  LR- | 1 externally validated performance measure (46/1372) | EV | Very serious | NA | No | Yes | Yes, | No | No | 0.7 | **Very low** |
| Hamilton screening tool  AUC | 1 apparent performance measure (76/114) | AP | Very serious | NA | No | No | Yes | Yes | No | 91.0% (87.0% to 96.0%) | **Very low** |
| Hamilton screening tool (Xiang version)  Sensitivity | 1 apparent performance measure (46/1372) | AP | Very serious | NA | No | Yes | Yes | No | No | 82.6% (68.6% to 92.2%) | **Very low** |
| Hamilton screening tool (Xiang version)  Specificity | 1 apparent performance measure (46/1372) | AP | Very serious | NA | No | Yes | Yes | No | No | 37.2% (34.7% to 39.9%) | **Very low** |
| Hamilton screening tool (Xiang version)  LR+ | 1 apparent performance measure (46/1372) | AP | Very serious | NA | No | Yes | Yes | No | No | 1.32 | **Very low** |
| Hamilton screening tool (Xiang version)  LR- | 1 apparent performance measure (46/1372) | AP | Very serious | NA | No | Yes | Yes | No | No | 0.47 | **Very low** |
| Hermann model  Sensitivity | 1 apparent performance measure (30/62) | AP | Very serious | NA | No | Yes | Yes | Yes | No | 90% | **Very low** |
| Hermann model  Specificity | 1 apparent performance measure (30/62) | AP | Very serious | NA | No | Yes | Yes | Yes | No | 95% | **Very low** |
| Hermann model  LR+ | 1 apparent performance measure (30/62) | AP | Very serious | NA | No | Yes | Yes | No | No | 16.8 (5.6 to 50.8) | **Very low** |
| Hermann model  LR- | 1 apparent performance measure (30/62) | AP | Very serious | NA | No | Yes | Yes | No | No | 0.11 | **Very low** |
| Hermann model  AUC | 1 apparent performance measure (30/62) | AP | Very serious | NA | No | Yes | Yes | Yes | No | 98% | **Very low** |
| MASTER strategy 1  PPV | 1 apparent performance measure (133/185) | AP | Very serious | NA | No | Yes | Yes | No | No | 41.8% | **Very low** |
| MASTER strategy 2  Sensitivity | 1 externally validated performance measure (249/222) | EV | Very serious | NA | No | Yes | No | No | No | 67.1% | **Very low** |
| MASTER strategy 2  Specificity | 1 externally validated performance measure (249/222) | EV | Very serious | NA | No | Yes | No | Yes | No | 79.7% | **Low** |
| MASTER strategy 2  LR+ | 1 externally validated performance measure (249/222) | EV | Very serious | NA | No | Yes | No | No | No | 3.3 | **Very low** |
| MASTER strategy 2  LR- | 1 externally validated performance measure (249/222) | EV | Very serious | NA | No | Yes | No | No | No | 0.41 | **Very low** |
| MASTER without imaging  Sensitivity | 1 externally validated performance measure (107/154) | EV | Very serious | NA | Yes | No | Yes | No | No | 64% | **Very low** |
| MASTER without imaging  Specificity | 1 externally validated performance measure (107/154) | EV | Very serious | NA | Yes | No | Yes | No | No | 76% | **Very low** |
| MASTER without imaging  LR+ | 1 externally validated performance measure (107/154) | EV | Very serious | NA | Yes | No | Yes | No | No | 2.68 | **Very low** |
| MASTER without imaging  LR- | 1 externally validated performance measure (107/154) | EV | Very serious | NA | Yes | No | Yes | No | No | 0.47 | **Very low** |
| MASTER modified  Sensitivity | 1 apparent performance measure (92/3785) | AP | Very serious | NA | No | No | Yes | No | No | 36.7% | **Very low** |
| MASTER modified  Specificity | 1 apparent performance measure (92/3785) | AP | Very serious | NA | No | No | Yes | Yes | No | 87.7% | **Very low** |
| MASTER modified  LR+ | 1 apparent performance measure (92/3785) | AP | Very serious | NA | No | No | Yes | No | No | 3.0 | **Very low** |
| MASTER modified  LR- | 1 apparent performance measure (92/3785) | AP | Very serious | NA | No | No | Yes | No | No | 0.7 | **Very low** |
| MASTER modified  AUC | 1 apparent performance measure (92/3785) | AP, | Very serious | NA | No | No | Yes | No | No | 62.2% | **Very low** |
| OptiRef  Sensitivity | 1 apparent performance measure (35/145) | AP | Very serious | NA | Yes | No | Yes | Yes | No | 100% | **Very low** |
| OptiRef  Specificity | 1 apparent performance measure (35/145) | AP | Very serious | NA | Yes | No | Yes | No | No | 12% | **Very low** |
| OptiRef  LR+ | 1 apparent performance measure (35/145) | AP | Very serious | NA | Yes | No | Yes | No | No | 1.14 | **Very low** |
| OptiRef  LR- | 1 apparent performance measure (35/145) | AP | Very serious | NA | Yes | No | Yes | No | No | 0.01 | **Very low** |
| Passalent screening tool  Sensitivity | 1 apparent performance measure (63/342) | AP | Very serious | NA | No | Yes | Yes | No | No | 73.0% (65.0% to 80.0%) | **Very low** |
| Passalent screening tool  Specificity | 1 apparent performance measure (63/342) | AP | Very serious | NA | No | Yes | Yes | No | No | 44.0% (38.0% to 50.0%) | **Very low** |
| Passalent screening tool  LR+ | 1 apparent performance measure (63/342) | AP | Very serious | NA | No | Yes | Yes | No | No | 1.3 | **Very low** |
| Passalent screening tool  LR- | 1 apparent performance measure (63/342) | AP | Very serious | NA | No | Yes | Yes | No | No | 0.61 | **Very low** |
| RADAR strategy 1  PPV | 1 externally validated performance measure (8/20) | EV | Very serious | NA | No | Yes | Yes | No | No | 28.5% | **Very low** |
| RADAR strategy 2  Sensitivity | 1 externally validated performance measure (249/222) | EV | Very serious | NA | No | Yes | Yes | No | No | 75.5% | **Very low** |
| RADAR strategy 2  Specificity | 1 externally validated performance measure (249/222) | EV | Very serious | NA | No | Yes | Yes | Yes | No | 75.2% | **Very low** |
| RADAR strategy 2  LR+ | 1 externally validated performance measure (249/222) | EV | Very serious | NA | No | Yes | Yes | No | No | 3.0 | **Very low** |
| RADAR strategy 2  LR- | 1 externally validated performance measure (249/222) | EV | Very serious | NA | No | Yes | Yes | No | No | 0.33 | **Very low** |
| RADAR without imaging  Sensitivity | 1 externally validated performance measure (107/154) | EV | Very serious | NA | No | No | Yes | No | No | 79% | **Very low** |
| RADAR without imaging  Specificity | 1 externally validated performance measure (107/154) | EV | Very serious | NA | No | No | Yes | No | No | 63% | **Very low** |
| RADAR without imaging  LR+ | 1 externally validated performance measure (107/154) | EV | Very serious | NA | No | No | Yes | No | No | 2.12 | **Very low** |
| RADAR without imaging  LR- | 1 externally validated performance measure (107/154) | EV | Very serious | NA | No | No | Yes | No | No | 0.33 | **Very low** |
| RADAR 2/3  Sensitivity | Meta – analysis of 2 externally validated performance measures (356/376) | EV | Very serious | No | No | Yes | No | No | No | 50.7% (43.0% to 58.4%) | **Very low** |
| RADAR 2/3  Specificity | Meta – analysis of 2 externally validated performance measures (356/376) | EV | Very serious | No | No | No | No | Yes | No | 81.6% (77.4% to 85.2%) | **Moderate** |
| RADAR 2/3  LR+ | Meta – analysis of 2 externally validated performance measures (356/376) | EV | Very serious | No | No | Yes, | No | No | No | 2.76 (2.13 to 3.59) | **Very low** |
| RADAR 2/3  LR- | Meta – analysis of 2 externally validated performance measures (356/376) | EV | Very serious | No | No | Yes | No | No | No | 0.60 (0.51 to 0.71) | **Very low** |
| RADAR by Bentin 1/3  PPV | 1 apparent performance measure (19/54) | AP | Very serious | NA | No | Yes | Yes | No | No | 26.0% | **Very low** |
| RADAR by Bentin 2/6  PPV | 1 apparent performance measure (19/54) | AP | Very serious | NA | No | Yes | Yes | No | No | 36.9% | **Very low** |
| SUSPECT  Sensitivity | 1 apparent performance measure (37/48) | AP | Very serious | NA | No | Yes | Yes | No | No | 59.4% | **Very low** |
| SUSPECT  Specificity | 1 apparent performance measure (37/48) | AP | Very serious | NA | No | Yes | Yes | No | No | 62.5% | **Very low** |
| SUSPECT  LR+ | 1 apparent performance measure (37/48) | AP | Very serious | NA | No | Yes | Yes | No | No | 1.58 | **Very low** |
| SUSPECT  LR- | 1 apparent performance measure (37/48) | AP | Very serious | NA | No | Yes | Yes | No | No | 0.65 | **Very low** |
| Weisman model  Sensitivity | 1 internally validated performance measure (43/92) | IV | Very serious | NA | No | Yes | Yes | No | No | 67.4% | **Very low** |
| Weisman model  Specificity | 1 internally validated performance measure (43/92) | IV | Very serious | NA | No | Yes | Yes | No | No | 94.6% | **Very low** |
| Weisman model  LR+ | 1 internally validated performance measure (43/92) | IV | Very serious | NA | No | Yes | Yes | No | No | 12.48 | **Very low** |
| Weisman model  LR- | 1 internally validated performance measure (43/92) | IV | Very serious | NA | No | Yes | Yes | No | No | 0.34 | **Very low** |
| Weisman model  AUC | 1 apparent performance measure (102/214) | AP | Very serious | NA | No | Yes | Yes | Yes | No | 94% | **Very low** |
