# Supplementary material for: Performance of referral strategies for patients with suspected axial spondyloarthritis: a systematic review with meta-analysis
Source: eClinicalMedicine. 2026 Aug 26;99:104167. doi: 10.1016/j.eclinm.2026.104167 (PMC13543815; doi:10.1016/j.eclinm.2026.104167)
Supplement: Supplementary file 10 [file mmc8.docx]

**Supplementary material 8 – Risk of bias of the performance metrics**

| **Referral strategy** | **Author and type of comparison** | **Type of performance** | **Risk of bias** | | | | **Applicability** | | | **Overall** | |
| --- | --- | --- | --- | --- | --- | --- | --- | --- | --- | --- | --- |
|  |  |  | **Participants** | **Predictors** | **Outcome** | **Analysis** | **Participants** | **Predictors** | **Outcome** | **Risk of bias** | **Applicability** |
| ASAS referral criteria | Abawi (2017) Rheumatologist’s diagnosis | EV | **+** | **-** | **-** | **-** | **+** | **+** | **+** | **-** | **+** |
|  | Abawi (2017) ASAS criteria | EV | **+** | **-** | **-** | **-** | **+** | **+** | **+** | **-** | **+** |
|  | Maskymowych (2025) (SAAPIC-1)  Rheumatologists’ diagnosis | EV | **+** | **-** | **-** | **-** | **+** | **+** | **+** | **-** | **+** |
|  | Maskymowych (2025) (SAAPIC-2)  Rheumatologists’ diagnosis | EV | **+** | **-** | **-** | **-** | **+** | **+** | **+** | **-** | **+** |
| ASAS referral criteria by Sepriano | Sepriano (2019) Rheumatologists’ diagnosis (consensus between three clinicians) | AP | **+** | **+** | **?** | **-** | **?** | **+** | **+** | **-** | **?** |
| ASAS referral criteria by Ziade | Ziade (2023)  Rheumatologist’s diagnosis | AP | **+** | **-** | **-** | **-** | **+** | **+** | **+** | **-** | **+** |
| A-tool | Danve (2025)  Rheumatologists’ diagnosis | AP | **+** | **-** | **-** | **-** | **?** | **+** | **+** | **-** | **?** |
| AWARE ≥ 1/5 | Braun (2019)  Rheumatologist’s diagnosis | AP | **-** | **+** | **-** | **-** | **+** | **+** | **+** | **-** | **+** |
| AWARE ≥ 2/5 - Braun IBP | Abawi (2017) Rheumatologist’s diagnosis | EV | **+** | **-** | **-** | **-** | **+** | **+** | **+** | **-** | **+** |
|  | Abawi (2017) ASAS criteria | EV | **+** | **-** | **-** | **-** | **+** | **+** | **+** | **-** | **+** |
|  | Braun (2011)  Rheumatologist’s diagnosis | AP | **-** | **+** | **-** | **-** | **?** | **+** | **+** | **-** | **?** |
|  | Braun (2019)  Rheumatologist’s diagnosis | EV | **-** | **+** | **-** | **-** | **+** | **+** | **+** | **-** | **+** |
|  | Sepriano (2019) Rheumatologists’ diagnosis (consensus between three clinicians) | EV | **+** | **+** | **?** | **-** | **?** | **+** | **+** | **-** | **?** |
|  | Ziade (2023)  Rheumatologist’s diagnosis | EV | **+** | **-** | **-** | **-** | **+** | **+** | **+** | **-** | **+** |
| AWARE ≥ 3/5 | Braun (2019)  Rheumatologist’s diagnosis | AP | **-** | **+** | **-** | **-** | **+** | **+** | **+** | **-** | **+** |
| Baraliakos combination I | Baraliakos (2020)  Rheumatologist’s diagnosis | AP | **+** | **-** | **-** | **-** | **+** | **+** | **+** | **-** | **+** |
| Baraliakos combination II | Baraliakos (2020)  Rheumatologist’s diagnosis | AP | **+** | **-** | **-** | **-** | **+** | **+** | **+** | **-** | **+** |
| Baraliakos combination III | Baraliakos (2020)  Rheumatologist’s diagnosis | AP | **+** | **-** | **-** | **-** | **+** | **+** | **+** | **-** | **+** |
| Brandt I | Abawi (2017) Rheumatologist’s diagnosis | EV | **+** | **-** | **-** | **-** | **+** | **+** | **+** | **-** | **+** |
|  | Abawi (2017) ASAS criteria | EV | **+** | **-** | **-** | **-** | **+** | **+** | **+** | **-** | **+** |
|  | Sepriano (2019) Rheumatologists’ diagnosis (consensus between three clinicians) | EV | **+** | **+** | **?** | **-** | **?** | **+** | **+** | **-** | **?** |
|  | Ziade (2023)  Rheumatologist’s diagnosis | EV | **+** | **-** | **-** | **-** | **+** | **+** | **+** | **-** | **+** |
| Brandt I plus imaging | Brandt (2007)  Rheumatologist’s diagnosis | AP | **+** | **-** | **-** | **-** | **+** | **+** | **+** | **-** | **+** |
|  | Proft (2020)  Rheumatologist’s diagnosis | EV | **+** | **-** | **-** | **-** | **?** | **+** | **+** | **-** | **?** |
| Brandt II | Abawi (2017) Rheumatologist’s diagnosis | EV | **+** | **-** | **-** | **-** | **+** | **+** | **+** | **-** | **+** |
|  | Abawi (2017) ASAS criteria | EV | **+** | **-** | **-** | **-** | **+** | **+** | **+** | **-** | **+** |
|  | Sepriano (2019) Rheumatologists’ diagnosis (consensus between three clinicians) | EV | **+** | **+** | **?** | **-** | **?** | **+** | **+** | **-** | **?** |
|  | Ziade (2023)  Rheumatologist’s diagnosis | EV | **+** | **-** | **-** | **-** | **+** | **+** | **+** | **-** | **+** |
| Brandt III | Abawi (2017) Rheumatologist’s diagnosis | EV | **+** | **-** | **-** | **-** | **+** | **+** | **+** | **-** | **+** |
|  | Abawi (2017) ASAS criteria | EV | **+** | **-** | **-** | **-** | **+** | **+** | **+** | **-** | **+** |
|  | Sepriano (2019) Rheumatologists’ diagnosis (consensus between three clinicians) | EV | **+** | **+** | **?** | **-** | **?** | **+** | **+** | **-** | **?** |
|  | Ziade (2023)  Rheumatologist’s diagnosis | EV | **+** | **-** | **-** | **-** | **+** | **+** | **+** | **-** | **+** |
| Braun 2-step | Abawi (2017) Rheumatologist’s diagnosis | EV | **+** | **-** | **-** | **-** | **+** | **+** | **+** | **-** | **+** |
|  | Abawi (2017) ASAS criteria | EV | **+** | **-** | **-** | **-** | **+** | **+** | **+** | **-** | **+** |
|  | Braun (2013)  Rheumatologist’s diagnosis | AP | **+** | **+** | **-** | **-** | **+** | **+** | **+** | **-** | **+** |
| Braun 2-step modified | Sepriano (2019) Rheumatologists’ diagnosis (consensus between three clinicians) | AP | **+** | **+** | **?** | **-** | **?** | **+** | **+** | **-** | **?** |
| Braun 2-step alt | Abawi (2017) Rheumatologist’s diagnosis | EV | **+** | **-** | **-** | **-** | **+** | **+** | **+** | **-** | **+** |
|  | Abawi (2017) ASAS criteria | EV | **+** | **-** | **-** | **-** | **+** | **+** | **+** | **-** | **+** |
| CaFaSpA | Van Hoeven (2014)  ASAS crtieria | IV | **+** | **+** | **-** | **-** | **+** | **+** | **+** | **-** | **+** |
|  | Van Hoeven (2015)  ASAS criteria | EV | **+** | **+** | **-** | **-** | **+** | **+** | **+** | **-** | **+** |
| CaFaSpA ≥ 1 | Abawi (2017) Rheumatologist’s diagnosis | EV | **+** | **-** | **-** | **-** | **+** | **+** | **+** | **-** | **+** |
|  | Abawi (2017) ASAS criteria | EV | **+** | **-** | **-** | **-** | **+** | **+** | **+** | **-** | **+** |
|  | Ziade (2023)  Rheumatologist’s diagnosis | EV | **+** | **-** | **-** | **-** | **+** | **+** | **+** | **-** | **+** |
| CaFaSpA ≥ 1.5 | Sepriano (2019) Rheumatologists’ diagnosis (consensus between three clinicians) | AP | **+** | **+** | **?** | **-** | **?** | **+** | **+** | **-** | **?** |
| CaFaSpA ≥ 2 | Abawi (2017) Rheumatologist’s diagnosis | EV | **+** | **-** | **-** | **-** | **+** | **+** | **+** | **-** | **+** |
|  | Abawi (2017) ASAS criteria | EV | **+** | **-** | **-** | **-** | **+** | **+** | **+** | **-** | **+** |
| Calin’s criteria for IBP | Abawi (2017) Rheumatologist’s diagnosis | EV | **+** | **-** | **-** | **-** | **+** | **+** | **+** | **-** | **+** |
|  | Abawi (2017) ASAS criteria | EV | **+** | **-** | **-** | **-** | **+** | **+** | **+** | **-** | **+** |
|  | Hermann (2009)  Rheumatologist’s diagnosis | AP | **+** | **-** | **-** | **-** | **+** | **+** | **+** | **-** | **+** |
|  | Sepriano (2019) Rheumatologists’ diagnosis (consensus between three clinicians) | EV | **+** | **+** | **?** | **-** | **?** | **+** | **+** | **-** | **?** |
|  | Ziade (2023)  Rheumatologist’s diagnosis | EV | **+** | **-** | **-** | **-** | **+** | **+** | **+** | **-** | **+** |
| EpiReumaPt | Sepriano (2019) Rheumatologists’ diagnosis (consensus between three clinicians) | AP | **+** | **+** | **?** | **-** | **?** | **+** | **+** | **-** | **?** |
| Hamilton screening tool | Hamilton (2023)  Modified NY citeria | AP | **+** | **-** | **-** | **-** | **+** | **+** | **+** | **-** | **+** |
|  | Xiang (2022)  Rheumatologist’s diagnosis | EV | **+** | **-** | **-** | **-** | **+** | **+** | **+** | **-** | **+** |
| Hamilton screening tool (Xiang version) | Xiang (2022)  Rheumatologist’s diagnosis | AP | **+** | **-** | **-** | **-** | **+** | **+** | **+** | **-** | **+** |
| Hermann model | Hermann (2009)  Rheumatologist’s diagnosis | AP | **+** | **-** | **-** | **-** | **+** | **+** | **+** | **-** | **+** |
| MASTER strategy 1 | Poddubnyy (2011)  Rheumatologist’s diagnosis | AP | **+** | **+** | **-** | **-** | **+** | **+** | **+** | **-** | **+** |
| MASTER strategy 2 | Poddubnyy (2011)  Rheumatologist’s diagnosis | AP | **+** | **+** | **-** | **-** | **+** | **+** | **+** | **-** | **+** |
|  | Ziade (2023)  Rheumatologist’s diagnosis | EV | **+** | **-** | **-** | **-** | **+** | **+** | **+** | **-** | **+** |
| MASTER without imaging | Abawi (2017) Rheumatologist’s diagnosis | EV | **+** | **-** | **-** | **-** | **?** | **+** | **+** | **-** | **?** |
|  | Abawi (2017) ASAS criteria | EV | **+** | **-** | **-** | **-** | **+** | **+** | **+** | **-** | **+** |
| MASTER modified | Sepriano (2019) Rheumatologists’ diagnosis (consensus between three clinicians) | AP | **+** | **+** | **?** | **-** | **+** | **+** | **+** | **-** | **+** |
| OptiRef | Proft (2020)  Rheumatologist’s diagnosis | EV | **+** | **-** | **-** | **-** | **?** | **+** | **+** | **-** | **?** |
| Passalent screening tool | Passalent (2022)  Rheumatologist’s diagnosis | AP | **+** | **+** | **-** | **-** | **+** | **+** | **+** | **-** | **+** |
| RADAR strategy 1 | Juanola (2023)  Rheumatologist’s diagnosis | EV | **+** | **+** | **-** | **-** | **+** | **+** | **+** | **-** | **+** |
|  | Sieper (2012)  Rheumatologist’s diagnosis | AP | **+** | **-** | **-** | **-** | **+** | **+** | **+** | **-** | **+** |
| RADAR strategy 2 | Juanola (2023)  Rheumatologist’s diagnosis | EV | **+** | **+** | **-** | **-** | **+** | **+** | **+** | **-** | **+** |
|  | Sieper (2012)  Rheumatologist’s diagnosis | AP | **+** | **-** | **-** | **-** | **+** | **+** | **+** | **-** | **+** |
|  | Ziade (2023)  Rheumatologist’s diagnosis | EV | **+** | **-** | **-** | **-** | **+** | **+** | **+** | **-** | **+** |
| RADAR without imaging | Abawi (2017) Rheumatologist’s diagnosis | EV | **+** | **-** | **-** | **-** | **+** | **+** | **+** | **-** | **+** |
|  | Abawi (2017) ASAS criteria | EV | **+** | **-** | **-** | **-** | **+** | **+** | **+** | **-** | **+** |
| RADAR 2/3 | Abawi (2017) Rheumatologist’s diagnosis | EV | **+** | **-** | **-** | **-** | **+** | **+** | **+** | **-** | **+** |
|  | Abawi (2017) ASAS criteria | EV | **+** | **-** | **-** | **-** | **+** | **+** | **+** | **-** | **+** |
|  | Ziade (2023)  Rheumatologist’s diagnosis | EV | **+** | **-** | **-** | **-** | **+** | **+** | **+** | **-** | **+** |
| RADAR by Bentin 1/3 | Bentin (2012)  Rheumatologist’s diagnosis | AP | **+** | **-** | **-** | **-** | **+** | **+** | **+** | **-** | **+** |
| RADAR by Bentin 2/6 | Bentin (2012)  Rheumatologist’s diagnosis | AP | **+** | **-** | **-** | **-** | **+** | **+** | **+** | **-** | **+** |
| SUSPECT | Tant (2017)  Rheumatologist diagnosis | AP | **+** | **-** | **-** | **-** | **+** | **+** | **+** | **-** | **+** |
| Weisman model | Weisman (2010)  NI | AP | **-** | **+** | **-** | **-** | **+** | **+** | **+** | **-** | **+** |
|  | Weisman (2010)  NI | IV | **-** | **+** | **-** | **-** | **+** | **+** | **+** | **-** | **+** |

AP: apparent performance, IV: internally validated performance, EV: externally validated performance, NI: not indicated.
